# Recent Advances in Active Infrared Thermography for Non-Destructive Testing of Aerospace Components

**DOI:** 10.3390/s18020609

**Published:** 2018-02-16

**Authors:** Francesco Ciampa, Pooya Mahmoodi, Fulvio Pinto, Michele Meo

**Affiliations:** Materials and Structures Centre (MAST), Department of Mechanical Engineering, University of Bath, Bath BA2 7AY, UK; P.Mahmoodi@bath.ac.uk (P.M.); f.pinto@bath.ac.uk (F.P.); m.meo@bath.ac.uk (M.M.)

**Keywords:** non-destructive evaluation, infrared thermography, material damage, composite materials, smart materials, aerospace structures

## Abstract

Active infrared thermography is a fast and accurate non-destructive evaluation technique that is of particular relevance to the aerospace industry for the inspection of aircraft and helicopters’ primary and secondary structures, aero-engine parts, spacecraft components and its subsystems. This review provides an exhaustive summary of most recent active thermographic methods used for aerospace applications according to their physical principle and thermal excitation sources. Besides traditional optically stimulated thermography, which uses external optical radiation such as flashes, heaters and laser systems, novel hybrid thermographic techniques are also investigated. These include ultrasonic stimulated thermography, which uses ultrasonic waves and the local damage resonance effect to enhance the reliability and sensitivity to micro-cracks, eddy current stimulated thermography, which uses cost-effective eddy current excitation to generate induction heating, and microwave thermography, which uses electromagnetic radiation at the microwave frequency bands to provide rapid detection of cracks and delamination. All these techniques are here analysed and numerous examples are provided for different damage scenarios and aerospace components in order to identify the strength and limitations of each thermographic technique. Moreover, alternative strategies to current external thermal excitation sources, here named as material-based thermography methods, are examined in this paper. These novel thermographic techniques rely on thermoresistive internal heating and offer a fast, low power, accurate and reliable assessment of damage in aerospace composites.

## 1. Infrared Thermography for Aerospace Applications

**Figure 1:**
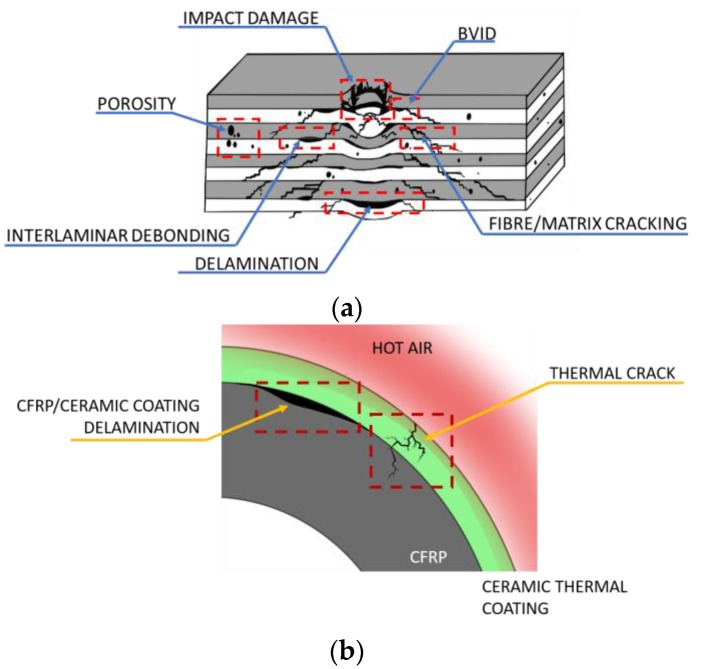

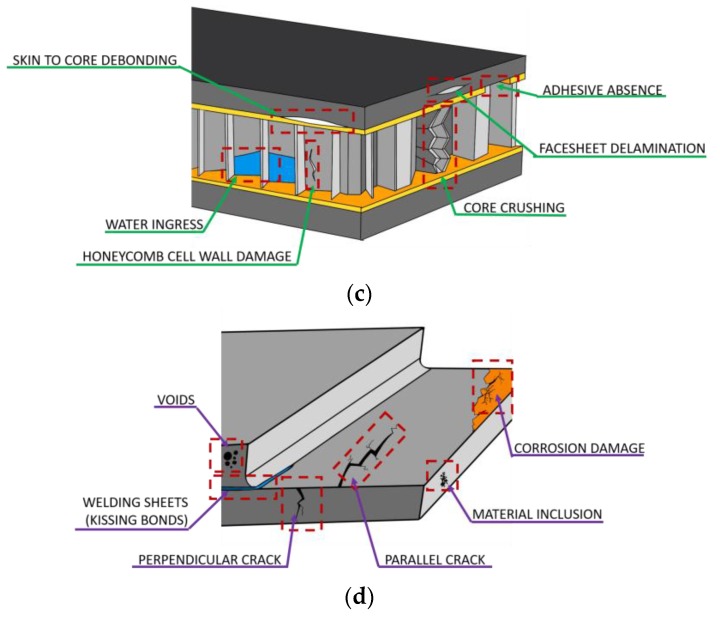
Illustration of typical material defects monitored by IRT for composite aircraft and spacecraft structures (**a**); jet engine turbine blades (**b**); honeycomb panels (**c**) and metallic aircraft and spacecraft components (**d**).

Infrared thermography (IRT) is a rapid and accurate non-destructive evaluation (NDE) technique that is widely used for the inspection of large aerospace components such as aircraft and helicopters’ primary and secondary structures, aero-engine parts, spacecraft components and its subsystems. IRT is generally applied to different materials including aluminium, composites and hybrid fibre metal laminates such a glass laminate aluminium reinforced epoxy (GLARE) and carbon reinforced aluminium laminate (CARALL) [1]. Whilst aluminium is today the primary material used in the aerospace industry, composite laminates, such as carbon fibre reinforced polymers (CFRP) and glass fibre reinforced polymers (GFRP), have been increasingly used due their excellent thermal-mechanical properties such as low weight, high specific strength and stiffness, corrosion resistant and thermal insulation [2,3,4]. It is estimated that composite components constitute to about 50% by weight of the current generation of aircraft and are used for engine casings, wing sections, tail plane, fuselage and control surfaces [5]. Typical damage scenarios in aerospace components include corrosion cracking, fatigue cracks, barely visible impact damage (BVID), delaminations, disbonding, matrix/fibre cracking, voids and core crushing. These types of material defects may occur at any stage of the component’s cycle life, from manufacturing to in-service and maintenance operations [6]. IRT has proven to be an effective tool to detect and, in many cases, quantify these material flaws. The most common materials and types of damage in aerospace components that can be monitored with IRT are reported in Table 1 and illustrated in Figure 1 [7,8,9,10,11].

IRT is generally classified as “passive” (stationary) and “active” (non-stationary) thermography [12,13]. Passive IRT is typically used for materials that are not in thermal equilibrium and have a different temperature from the environment in which they operate. Passive thermography is suitable for cyclic loading as the resulting hysteretic heat allows for time-dependent temperature variations that can be monitored with an infrared (IR) camera. As an example from Montesano et al. [14], passive thermography can be used for the detection of water ingress just after aircraft landing due to a significant temperature difference between the aircraft and water. 

Active IRT, on the other hand, employs external excitation sources such as optical radiation (e.g., halogen heat lamps and laser), electromagnetic stimulation (induced eddy currents and microwaves), mechanical ultrasonic waves and material enabled features in composites in order to generate heat in the component under inspection [15,16]. In Active IRT, temperature gradients responses at the medium surface are recorded and analysed by an IR camera to provide information about the integrity of the structure. Indeed, as thermal waves flow inside the sample by diffusion, the heat diffusion rate over a material defect will differ from the surrounding area and the resulting thermal response can be used to detect and quantify material damage [17]. For this reason, active IRT has very competitive costs compared to more sophisticated NDE equipment such as ultrasonic phased array and X-ray system (CT-Scan). Literature offers a vast review and classification of active IRT for different application fields and types of materials/damage [18,19]. However, a deep review including the principles, recent developments, strengths and limitations of active IRT thermography specifically for the aerospace industry is still missing and constitutes the main focus of this work. Table 2 summarises the main active IRT methods used in aerospace industry according to their physical principle and thermal sources, which will be analysed in this review. 

## 2. Optically Stimulated Thermography

**Figure 2:**
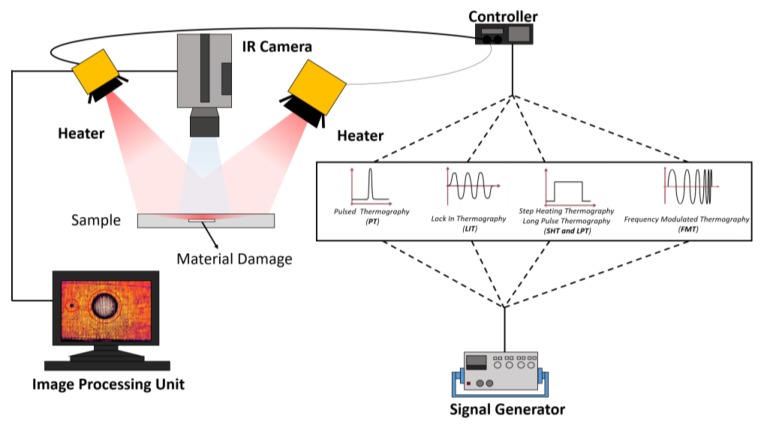
Schematic diagram of optically stimulated thermography (OST).

Depending on the type of external optical heat source used to generate thermal waves, the most common methods used in optically stimulated thermography (OST) for aerospace applications are pulsed thermography (PT), lock-in thermography (LIT), step heating thermography (SHT) and long pulse thermography (LPT), frequency modulated thermography (FMT), laser-spot thermography (LST) and laser-line thermography (LLT) [35,36,37,38]. Figure 2 shows the experimental setup of OST for different optical excitation sources.

Data obtained by an IR camera in OST are often contaminated by different noise sources such as external reflections, variations in the optical properties of the specimen and non-uniform heating. These noise effects in thermal images produce abnormal thermal patterns and make the detection of damage more complex. A number of signal processing techniques has been developed to reduce the noise in thermal images, thus enhancing the damage detection and quantification capabilities of each OST method [39,40]. An extensive review of signal processing methods for IRT data was recently provided by [41]. Table 3 reports a summary of the most used signal processing techniques for OST and their basic principles.

### 2.1. Pulsed Thermography 

PT is a rapid NDE process that uses a high intensity pulse of light to heat the surface of a test piece via the photothermal effect [41]. Very short duration flashes (ranging between 0.1 and 50 ms) are typically employed, depending on the sample thermal properties and defect depths. The temperature gradients of the sample surface are monitored by an IR camera with a computer system to quantify the material damage [53]. Sub-surface defects reduce the conduction of heat away from the surface, decreasing the surface cooling rate compared to that of a non-defective region. The transient temperature field *T*(*z*,*t*) in PT is obtained as solution of the following inhomogeneous one-dimensional (1D) heat equation [54]:

(1) $$\frac{\partial^{2}T\left(z,t\right)}{\partial z^{2}}-\frac{1}{\alpha }\frac{\partial T\left(z,t\right)}{\partial t}=-\frac{g\left(z,t\right)}{k}\;t>0$$

where *α* = *k/ρc* is the thermal diffusivity (m^2^/s), with *k* the thermal conductivity (W/mK), *ρ* the density (kg/m^3^) and *c* the specific heat (J/kg K), *g*(*z,t*) = *Q*_0_*δ*(*z−z*)*δ*(*t−t*_0_) is the external impulsive heat source located in *z*_0_ = 0 and excited at time *t*_0_ = 0, with *Q*_0_ the source intensity per unit length (Jm^−1^) and *δ*(*z−z*)*δ*(*t−t*_0_) Dirac’s delta functions in space and time, respectively. According to Green’s theorem [55], to solve the linear inhomogeneous transient heat Equation (1), it is useful to define a Green space-time function that satisfies the homogeneous boundary conditions on the boundary of the material. Hence, the solution of the inhomogeneous heat Equation (1) can be expressed as follows:

(2) $$T\left(z,t\right)=\frac{Q_{0}}{2\zeta \sqrt{\pi t}}e^{-\frac{z^{2}}{4\alpha t}}$$

where $\xi =\sqrt{k\rho c}$ is the thermal effusivity (Ws^1/2^ m^−2^ K^−1^) of the material, which controls the reflection of heat from discontinuities. Equation (2) represents the temperature rise at a depth *z* beneath the surface at a time *t* after a uniform impulse of energy *Q*_0_. The surface thermal response is simply obtained from Equation (2) by posing *z* = 0. Assuming a region containing a delamination-like defect as a layer of thickness *d*, i.e., the depth of the defect below the surface, the impulsive thermal response *T_d_*(0,*t*) at the surface becomes [53]:

(3) $$T_{d}\left(0,t\right)=\frac{Q_{0}}{2\zeta \sqrt{\pi t}}\left\{1+2\sum_{n=1}^{\infty }\left[e^{-\frac{{\left(nd\right)}^{2}}{\alpha t}}-e^{-\frac{{\left(nL\right)}^{2}}{\alpha t}}\right]\right\}\left(1-e^{-\frac{{\left(D/2\right)}^{2}}{4\alpha t}}\right)$$

where, *L* is the thickness of the material and *D* is the diameter of the circular defect.

Pulsed Phase Thermography (PPT) is the phase analysis in frequency domain of PT and is a signal processing method that solves the problem of heating inhomogeneity as it is less sensitive to the non-uniformity of heating, surface irregularities and environmental reflections [56]. It can also image large components on airframes relatively fast [57]. Since PPT is widely used in the aerospace industry for the inspection of aerospace metallic and composite components [58], it can be considered as a valid alternative to time domain PT. Low frequencies, typically up to 2–3 Hz are used in PPT to assess deeper defects. The procedure to extract the phase from the thermal data *T*(*k*) is based on the Fourier Transform, which is computed according to the well-known formula [59]:

(4) T^n=∑n=1NT(k)exp(−j2πknN)=Ren+Imn

where *j* is an imaginary number, *n* design the modulation frequency increment, *N* is the total number of thermograms, and Re*_n_* and Im*_n_* are the real and imaginary parts of the Fourier Transform ${\hat{T}}_{n}$ of the thermal emissivity data. Hence, the phase *φ_n_* is simply obtained as *φ_n_* = tan^−1^(Im*_n_*/Re*_n_*). For PPT evaluation, the term “blind frequency” *f_b_* is often used as the limiting frequency at which the defect presents enough phase contrast to be detected on the phase spectrum. The “blind frequency” and the defect depth are typically correlated by the following formula [60]:

(5) $$d=C_{1}\sqrt{\frac{\alpha }{\pi f_{b}}}$$

where *C*_1_ is called a “correlation constant” and ranges between 1.5 and 2 [61]. 

**Figure 3:**
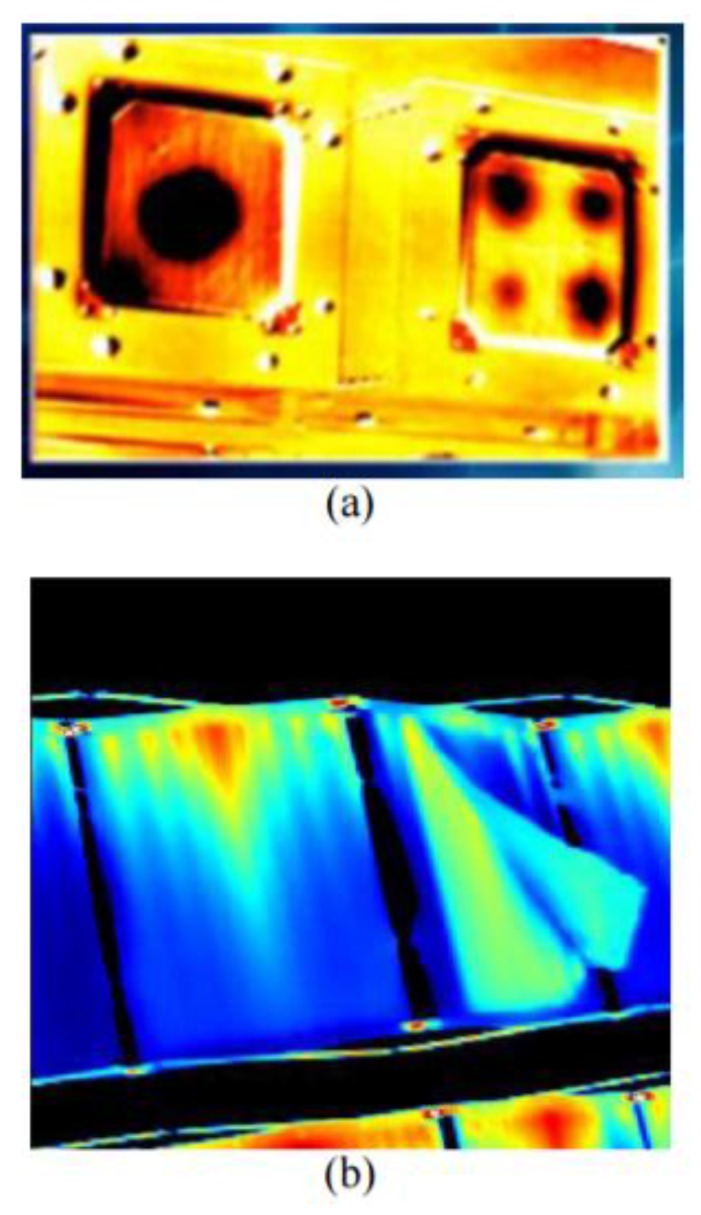
PT results obtained during EVA inspections of a CFRP component of the Space Shuttle (**a**) and a damaged ISS radiator (**b**), from [63].

PT can be applied to several types of aerospace materials such as composite, metallic and polymeric components [16,62]. In Cramer’s report [63], NASA used PT for on-orbit inspection of manned flight vehicles and the International Space Station (ISS). Figure 3 shows the PT results coupled with solar heating achieved by astronauts during extravehicular activity (EVA) inspections. In particular, it illustrates a PT inspection of both a CFRP component of the Space Shuttle with holes and a damaged ISS radiator. Black holes in Figure 3a correspond to pre-damaged carbon-carbon composite samples imaged by the EVA IR camera.

**Figure 4:**
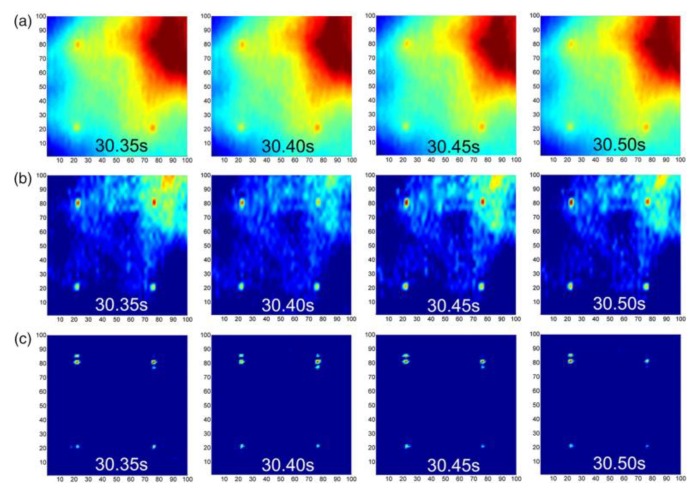
Comparison between PT thermal results from raw data (**a**); Differential Absolute Contrast (DAC) method (**b**) and Gapped Smoothing Algorithm (GSA) (**c**), with permission from [50].

Li et al. [50] assessed the performance of the Gapped Smoothing Algorithm (GSA, see Table 3) for damage detection on an aluminium plate and a glass fibre reinforced composite laminate containing different defects such flat-bottom holes, delamination and material impurities. The authors used a TIM-160 IR camera (MICRO-EPSILON, Ortenburg , Germany) and four heating lamps (240 V, 275 W) to generate heat. The numerical results on both aluminium and composite plates demonstrated that the proposed GSA method was robust, accurate and effective for detecting sub-surface flaws (Figure 4). 

Manhoar and Lanza di Scalea [64] developed and validated a three-dimensional (3D) analytical model to simulate the heat flow interaction with defects in a CFRP panel containing rectangular flat-bottom holes of different sizes. The authors found that experimental results were reasonably close to theoretical predictions. Montesano et al. [14] investigated the fatigue behaviour of a triaxially braided carbon fibre fabric (T650/35-6 K), with a 0°/±ϑ braid orientation, embedded in a thermosetting polyimide resin. A SC5000 IR camera (FLIR, Wilsonville OR, United States) with spatial resolution of 320 × 240 was used and heat was generated by mechanical loading. The authors elaborated a fatigue threshold value that was in excellent agreement with that obtained through a conventional PT experimental test. Guo and Vavilov [65] used PT for the detection of disbonds in the insulation of solid rocket motors made of a low-density rubber-like elastomer. The authors compared several data processing approaches based on the correlation technique and showed that the highest signal-to-noise ratio (SNR) appeared when choosing a reference point in a defect-free area of the sample. 

As reported in Table 3, advanced signal processing methods have been applied to PT in order to enhance the detection and quantification of material damage in aerospace components. Lopez et al. [51] used the Partial Least Square Thermography (PLST) method to optimise the processing of PT data on a CFRP composite with simulated defects. The experimental results, achieved with a FPA SBF125 IR camera (Santa Barbara, Lowell MA, USA) and two photographic flash lamps (FX60, Glamox, Borehamwood, UK) powered at 6.4 kJ, showed an increase in the SNR for 96% of defects after processing the images with the PLST technique. Zhao et al. [52] used the Coefficient Clustering Analysis (CSA) method coupled to PT, whilst Chang et al. [49] employed the Multi-dimensional Ensemble Empirical Decomposition (MEEMD) algorithm to quantify material damage in impacted CFRP composites. Pawar and Vavilov [48] used a 3D Normalisation Algorithm (3DNA) for PT to compensate the background non-uniformity in both damaged glass and carbon fibre reinforced composites. Tang et al. [66] combined the Markov and Principal Component Thermography (PCT) algorithm with PT in order to detect debonding in ceramic thermal barrier coatings for aerospace. The depth and diameter prediction error was about 4–10% for defects with a diameter–depth ratio of 1.2–4.0, and depth between 1.0 and 2.5 mm, which proved the effectiveness of the proposed method for a quantitative detection of material defects. Li et al. [67] compared the PT method with ultrasonic stimulated thermography (UST, see Section 3). The authors found that PT is more suitable for detecting delamination, especially with large areas, whilst UST is superior to detect small cracks such as matrix cracking and fibre breakage. The combination of the above two methods can greatly improve the capability to detect and evaluate impact damage in aerospace composites. 

**Figure 5:**
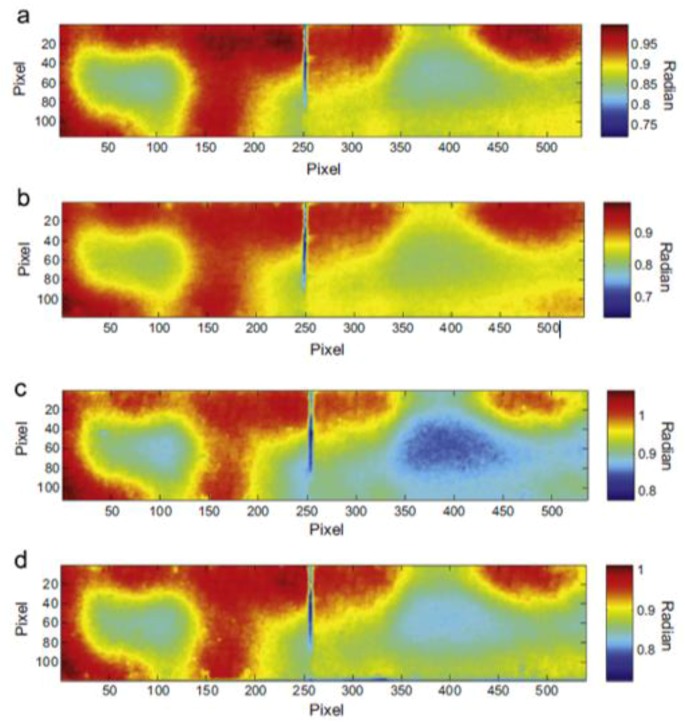
PPT phase results on a single lap joints with CFRP adherends undergone to different fatigue loading cycles, with permission from [57]. Each subfigure corresponds to the thermal phase image of the composite sample after 200 cycles (**a**); 400 cycles (**b**); 400 cycles and pretension (**c**) and 600 cycles (**d**).

Shin et al. [57] used PPT to detect the initiation and propagation of fatigue-induced damage in single lap joints with CFRP adherends (Figure 5). The authors developed a threshold technique that allowed the identification of fatigued samples with early stage material damage, thus preventing premature failures. Oswald-Tranta [68] provided a quantitative comparison between the time domain Thermographic Signal Reconstruction (TSR) method and the frequency domain PPT for an aluminium sample with flat bottom holes. The author found that whilst both signal-processing methods provide good SNR, the maximum of second derivative used in TSR allows an easier calculation of the defect depth compared to Equation (5) in PPT. Moreover, unlike PPT, TSR does not require any selection of a sound reference point.

### 2.2. Lock-In Thermography

**Figure 6:**
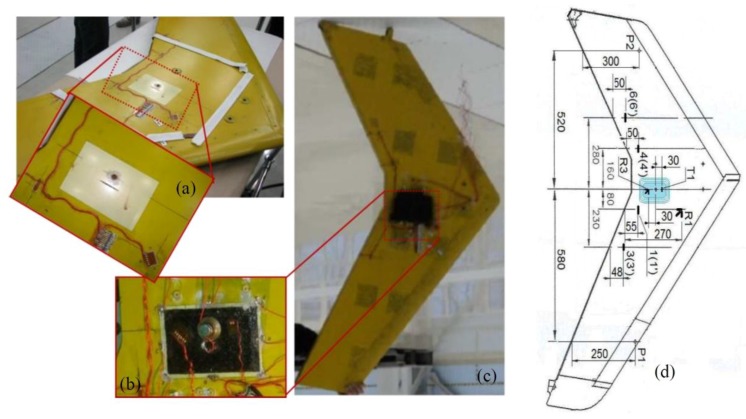
Illustration of the wing and its schematic representation analysed with LIT, from [73]. Unit measures in Figure 6d are in mm.

In LIT, an external periodic heat excitation is used to generate thermal waves within the sample. This is usually performed by employing sinusoidally modulated tungsten-halogen flood lamps [69]. Temperature variations at damage location are monitored with an IR camera and the magnitude images are computed for each heat-generating frequency by post-processing the recorded thermal data using the Fourier transform algorithm. Thermal images are recorded throughout the heating period that lasts for at least a full excitation cycle. LIT has the advantage of being less sensitive to the local variations of surface emissivity and continuous IR radiation once the incandescent lamp is switched off [17]. For a homogenous isotropic semi-infinite material whose surface is uniformly illuminated by a light beam of periodically modulated intensity *q_z_*= *Q*_0_[1+exp(jωt)]/2, with *ω* being the angular modulation frequency of the heat source, the temperature field can be obtained by solving the homogeneous 1D heat diffusion equation associated with Equation (1). The solution is expressed as follows [70]:

(6) $$T\left(z,t\right)=\frac{Q_{0}}{2\zeta \sqrt{\omega }}e^{-\frac{z}{\mu }}e^{j\left(\omega \;t-\frac{z}{\mu }\right)}$$

where $\mu =\;\sqrt{2\alpha /\omega }$ is the thermal diffusion length of the material. As it can be seen from Equation (6), thermal waves are very heavily damped with a constant decay equal to the thermal diffusion length. Moreover, thermal waves are characterised by a dispersive behaviour, with their phase velocity defined as $\nu =\omega \mu =\sqrt{2\alpha \omega }$. The response at the surface monitored by an IR camera can be obtained either from Equation (6) by posing *z* = 0, or as function of the thermal surface response using PT at the time *t =* 1 s [70]:

(7) $$T\left(0,t\right)=\frac{Q_{0}}{2\zeta s\sqrt{\omega }}$$

with *s* being the duration of the LIT experiment. Duan et al. [71] provided a quantitative evaluation of both LIT and PT for a set of aluminium foam specimens used for aerospace applications with flat-bottom holes. The authors found that although the post-processing methods of PT (including TSR and PCT, see Table 3) improve the thermal contrast for sub-surface and deeper defects, it is more difficult to analyse raw PT data due to non-uniform heating. Kordatos et al. [72] combined LIT and acoustic emissions measurements in order to determine the fatigue limit on dog-bone aluminium and cross-ply SiC/BMAS composite samples used in aircraft components. Grammatikos et al. analysed an aluminium wing structure with CFRP composite patches using LIT in order to assess the durability and efficiency of the patch repair during both maintenance (off-line) and service (on-line) conditions (Figure 6). 

**Figure 7:**
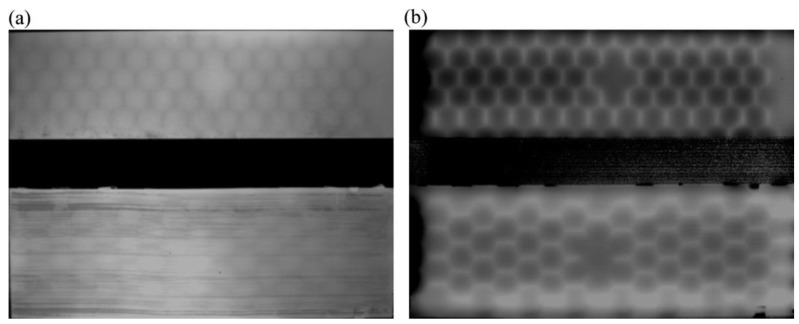
Ampligram (**a**) and phasegram (**b**) obtained with LIT testing of a titanium alloy honeycomb sandwich structure with disbond, with permission from [74].

Zhao et al. [74] analysed the phase variations of the surface temperature with LIT in order to investigate skin-to-core disbonds in a titanium alloy honeycomb structure (Figure 7). The authors also developed a three-dimensional finite element model of the titanium alloy honeycomb sandwich and found good agreement between numerical and experimental results. 

**Figure 8:**
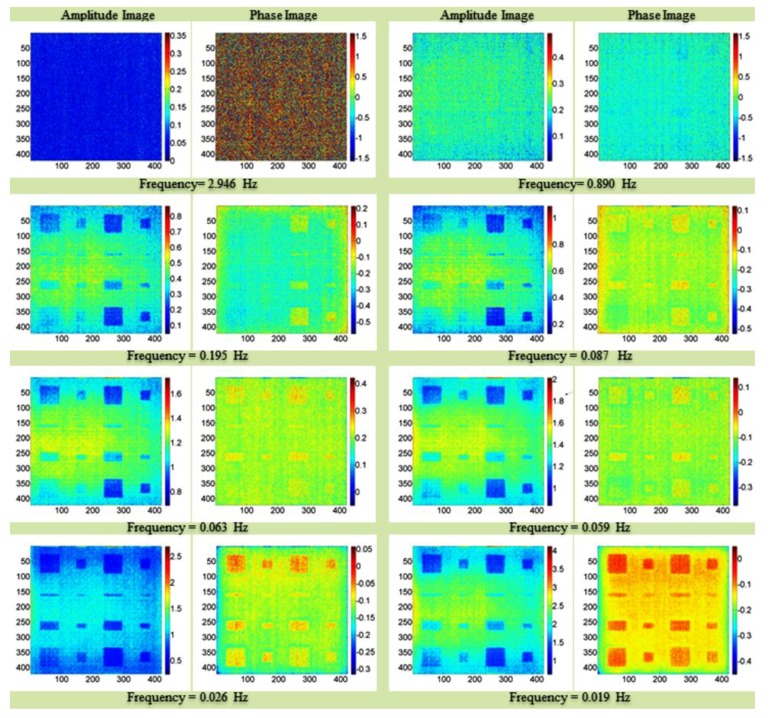
Lock-in thermography amplitude and phase images on a GFRP composite plate with artificial defects, with permission from [84].

A number of authors has recently used LIT for damage detection and quantification in aerospace composite components [75,76,77,78,79]. Junyan et al. [80] used LIT to quantify damage in SiC coated carbon-carbon composites used for the spacecraft re-entry thermal protection system. The same authors also developed an inverse approach applied to LIT in order to estimate the thermal diffusivities and sub-surface defect size of CFRP laminate composites [3]. Such inverse approach consisted of solving the objective function constructed from LIT phase images because of being less sensitive to non-uniform heating. A FLIR SC 7000 camera (FLIR, Wilsonville OR, United States) with a resolution of 320 × 256 was used for the experimental testing. De Angelis et al. [4] compared LIT with other NDE systems such as shearography, thermosonics and traditional ultrasounds in order to provide flaw detection across vulnerable surfaces of a Typhoon air-cooling inlet composite panel. The authors found that LIT can be used for both laboratory and/or full field applications of aeronautical composite components. Similarly, Ranjit et al. [81] provided a quantitative analysis of artificial defects in a GFRP composite plate of dimensions 300 × 300 × 1.4 mm by comparing LIT amplitude and phase images with shearograhy and linear ultrasounds (Figure 8). A FLIR SC465 IR camera with a resolution of 640 × 480 was used for the thermography testing. 

Meola et al. [82] used LIT to monitor impacted composite samples with different types of reinforcement (e.g., carbon and glass fibres) and matrix (e.g., thermoset and thermoplastic with a compatibilising agent). The authors found that although LIT is able to reveal different types of failures such as fibre breakage and delamination, it still fails to appraise the whole extension of the delaminated zone from the amplitude and phase images. Indeed, micro-crack and delamination can be confused with small material non-uniformities.

### 2.3. Step Heating and Long Pulse Thermography

In SHT and LPT a low intensity step-pulse (heat source) is applied for a long period (typically from milliseconds to few seconds), thus enabling a longer heating time to locate deeper defects. The main difference between the two techniques is that in SHT data are measured during the application of the step-pulse, whereas in LPT thermal signals are acquired during the cooling phase [23]. 

The temperature response *T*(0,*t*) measured at the surface of a homogeneous and semi-infinite medium is obtained as solution of the homogeneous 1D heat diffusion equation associated with Equation (1) by applying a step-pulse heating source Q_0_ [54]. This is expressed as follows:

(8) $$T\left(0,t\right)=\frac{2Q_{0}}{\zeta }\sqrt{\frac{t}{\pi }}$$

In the case of a material with finite thickness *L*, the surface temperature *T*(0,*t*) is given by [22,83]:

(9) $$T\left(0,t\right)=\frac{2Q_{0}}{\zeta }\sqrt{\frac{t}{\pi }}\left[1+\sqrt{\pi }\sum_{n=1}^{\infty }2ierfc\left(\frac{nL}{\sqrt{\alpha t}}\right)\right]$$

with $ierfc\left(x\right)=1/\left(\sqrt{\pi }\exp\left(-x^{2}\right)-x\cdot erfc\left(x\right)\right)$. Roche and Balageas [84] compared the thermal imaging results obtained with both SHT and PT in order to detect material damage located at different depth within a CFRP composite plate. The authors found that without any signal processing technique applied to both thermographic methods (i.e., during visual examination), SHT was able to reveal a surface defects but not in-depth damage. However, when TSR was applied to both SHT and PT, the last technique was better contrasted and allowed a better damage identification even for deepest defects. These results were also confirmed by Almond et al. [23], who applied SHT to both aluminium and composite samples with flat bottom holes of different dimensions and depth. The authors provided an analytical study of the SHT process, which revealed that the magnitude of defect image contrast is dependent on the thermal response rate of the material.

### 2.4. Frequency Modulated Thermography

FMT uses a non-stationary form of thermal excitation in which a low intensity frequency modulated input signal is used to generate thermal waves within the material [24]. Unlike LIT that uses a single excitation frequency for a fixed damage depth resolution, FMT can be used to detect material flaws located at various depths in the test sample [85]. The temperature field of intensity $q_{z}=Q_{0}\left[1+\exp\left[j\left(\omega t+\frac{\pi Bt^{2}}{\tau }\right)\right]/2]\right]$, with *τ* the duration of the frequency modulated heat cycle of bandwidth *B*, is obtained as solution of the homogeneous 1D heat diffusion equation associated with Equation (1). This is defined as follows [24]:

(10) $$T\left(z,t\right)=\frac{Q_{0}}{2\zeta \sqrt{\omega }}e^{-\frac{z}{\mu_{FM}}}e^{j\left(\omega t+\frac{\pi Bt^{2}}{\tau }-\frac{z}{\mu_{FM}}\right)}$$

with $\mu_{FM}=\sqrt{2\alpha /\left(\omega +\frac{2Bt}{\tau }\right)}$ the thermal diffusion length of the frequency modulated heating. Arora et al. [86] applied FMT on a CFRP sample with flat bottom holes and analysed the depth scanning performance using both time domain (magnitude) and frequency domain (phase) imaging data. The experimental results showed that phase retained its advantages of energy concentration, which led to enhanced depth scanning with improved resolution and sensitivity for defect detection. 

### 2.5. Laser-Spot Thermography and Laser-Line Thermography

**Figure 9:**
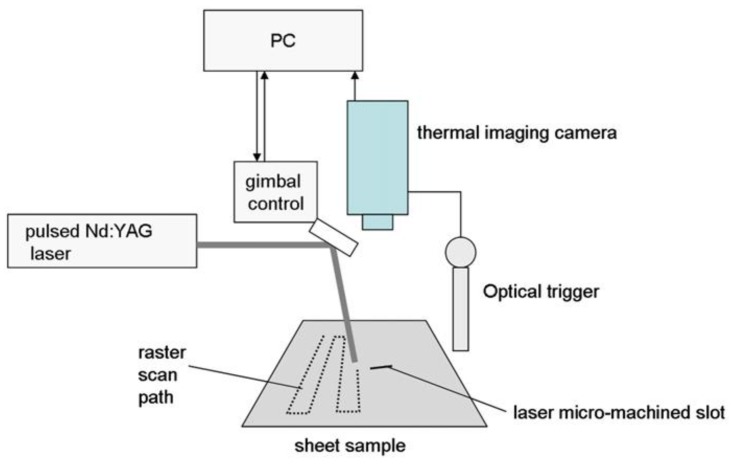
Experimental set-up for laser scanning thermography, with permission from [90].

The optical flash lamp heating process used for LIT, SHT and FMT produces a uniform area of heating across the surface of the test specimen. This results in a heat flow from the surface into the bulk, which limits the detected material flaw to delaminations lying in a plane parallel to the surface (interlaminar) or at the interfaces between a surface coating and its substrate. Hence, material damage that forms in planes perpendicular to the surface (intralaminar) is not detectable with traditional flash lamps [25]. Cracks of this type can be detected if the heating process is localised to a spot or a line on a surface. In both LST and LLT, damage can be identified either by a change in the shape of the heated spot or by an apparent increase in temperature [87]. A complete inspection of a test piece is achieved by processing thermal images collected during scanning the spot or line over the test piece surface. In the case of LLT, either the sample is moving with a constant speed passing in front of a fixed line heat source or vice versa [26]. The IR camera records the temperature distribution at the surface in a specified distance after the heating (cooling effect [88]). Figure 9 illustrates the schematic diagram for LST.

In LST, the temperature field *T*(*r,z,t*) after the laser spot is switched off is analytically obtained by convoluting a Gaussian shape round spot source (i.e., the laser pulse) with continuous heating. This is expressed as follows [54,89]:

(11) $$T\left(r,z,t\right)=\frac{I_{\max}a^{2}}{k}\sqrt{\frac{\alpha }{\pi }}\int_{0}^{t}\frac{p\left(t-t^{\prime }\right)e^{-\frac{z^{2}}{4\alpha t^{\prime }}}e^{-\frac{r^{2}}{4\alpha t^{\prime }+a^{2}}}}{\sqrt{t^{\prime }}\left(4\alpha t^{\prime }+a^{2}\right)}dt^{\prime }$$

where (*r,z*) are cylindrical coordinates with the origin on the surface at the centre of the irradiated spot, with $r=\sqrt{x^{2}+y^{2}}$, *I*_max_ and *a* are the maximum power density of the laser pulse and the radius of the laser beam, respectively, and *p*(*t*) is the normalised temporal profile of the laser pulse at the time *t*. The temperature response *T*(*r,*0*,t*) measured at the surface is obtained by posing *z* = 0 in Equation (11).

In LLT, a laser line source can be approximated to a Gaussian shape elliptical spot source with continuous heating and the temperature profile after cooling is given by [54]:

(12) $$T\left(x,y,z,t\right)=\frac{I_{\max}ab}{k}\sqrt{\frac{\alpha }{\pi }}\int_{0}^{t}\frac{p\left(t-t^{\prime }\right)e^{-\frac{z^{2}}{4\alpha t^{\prime }}}e^{-\frac{x^{2}}{4\alpha t^{\prime }+a^{2}}}e^{-\frac{y^{2}}{4\alpha t^{\prime }+b^{2}}}}{\sqrt{t^{\prime }\left(4\alpha t^{\prime }+a^{2}\right)\left(4\alpha t^{\prime }+b^{2}\right)}}dt^{\prime }$$

with *x*, *y* and *z* the Cartesian coordinates and *a* and *b* the laser beam radii. Similarly to Equation (11), the temperature response *T*(*x,y,*0*,t*) measured at the surface is obtained by posing *z* = 0 in Equation (12).

**Figure 10:**
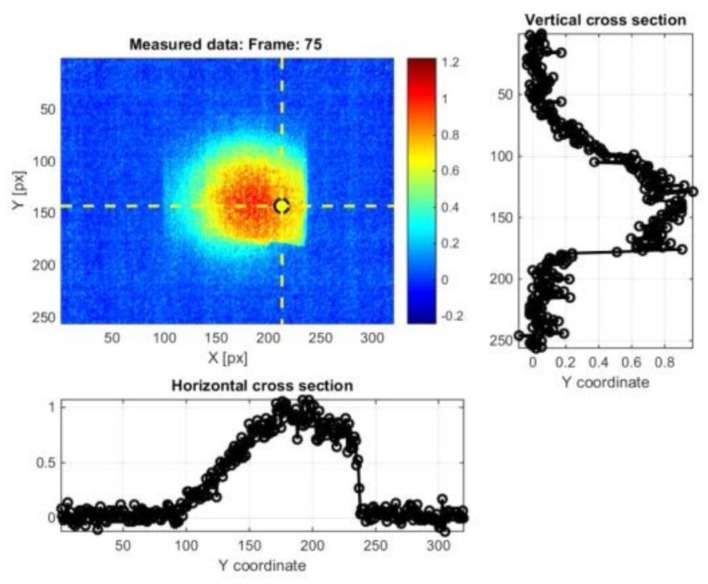
Laser-spot thermography result on fatigue damaged aluminium sample, from [93].

Ley et al. [90,91] used laser thermography to detect sub-surface defects in different composite materials commonly used in aerospace applications such as composite sandwich panels with artificial flaws (i.e., embedded Teflon patches) and real impact damage. The authors found good agreement between the laser thermography results and those obtained with ultrasonic C-Scan mesurements. Fedala et al. [92] combined LIT with a modulated laser excitation for the assessment of surface cracks in metallic turbine blades. Roemer et al. [93] compared LST with UST (see Section 3) to detect fatigue cracks on an aluminium sample (Figure 10). Khodayar et al. [94] used robotized Line Scan Thermography to inspect large composite materials, whilst Zhang et al. [95] used pulsed micro-laser line thermography to detect submillimeter porosity in CFRP composites. Fernandes et al. [96] employed a flying laser spot to heat a line region on a CFRP sample and assess the fiber orientation over that region. The non-contact nature and predispositions for automatization makes LST and LLT very promising for aerospace applications. Moreover, by simply combining IR cameras with zooming and focusing lenses, these techniques can be also used to identify micro-defects.

## 3. Ultrasonic Stimulated Thermography

**Figure 11:**
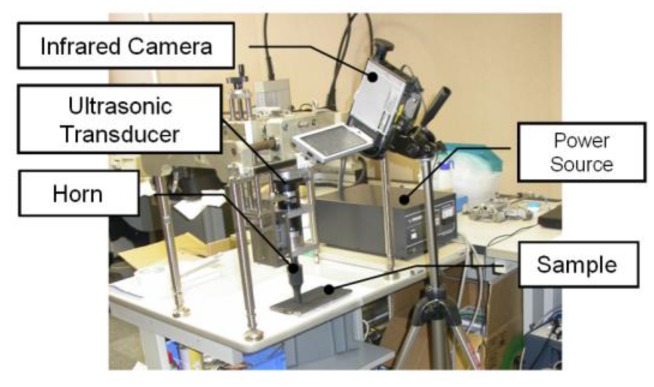
Experimental set-up of ultrasonic stimulated thermography, from [98].

**Figure 12:**
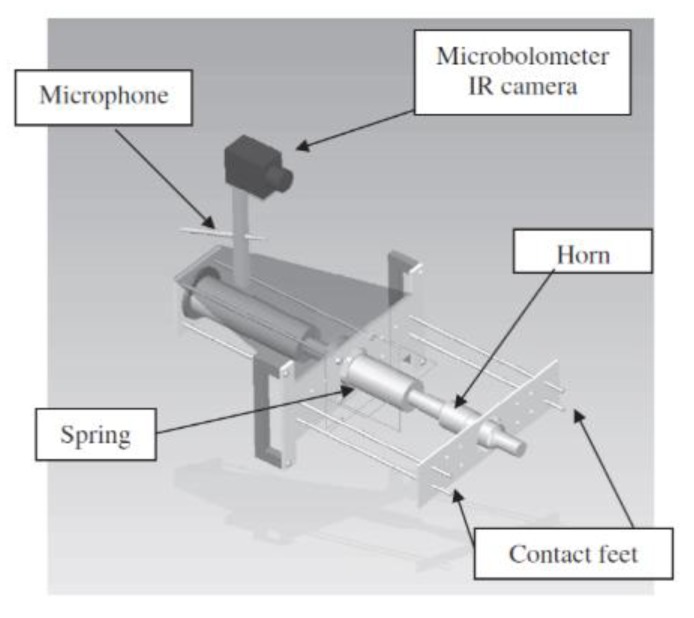
Experimental set-up of ultrasonic stimulated thermography, with permission from [98].

Optically Stimulated Thermography (OST) uses external optical flashes to heat the sample on its surface and then an IR camera to record the temperature decay curve. Although good results can be obtained for surface inspection, this technique may not be suitable for detecting in-depth damage, closed cracks and damage failures at the early stages. Indeed, the usage of external optical heat sources may limit the detection of defects to those located within a few millimetres from the material surface. In addition, the lack of a significant air gap between the cracked surfaces may not generate any significant variation of the local temperature needed for damage detection. UST (also known as thermosonics, sonic IR or vibro-thermography) is an alternative means of thermography that involves the generation of powerful vibrations in a test piece to cause frictional heating at crack surfaces. An IR camera with temperature resolution ranging between 20 and 40 mK is usually used to measure the frictional heating on the sample’s surface [97,98,99]. Figure 11 shows the experimental set-up for thermosonics. Elastic vibrations are generally produced by an ultrasonic plastic welding horn being pressed against a surface on the part under inspection (Figure 12). A pulse of high acoustic power (between 1 kW and 80 kW) in the 15–50 kHz frequency range is typically applied and the duration can vary from 30 to 200 ms.

Similarly to OST, depending on the shape of the ultrasonic driving signal, additional excitations can be used in thermosonics. These include “ultrasonic lock-in thermography” with continuous monochromatic elastic input [100] and “ultrasonic frequency modulated thermography” with chirp excitation signals [101]. Umar et al. [101] recently reported a review of earlier and later research on thermosonics for different application areas.

**Figure 13:**
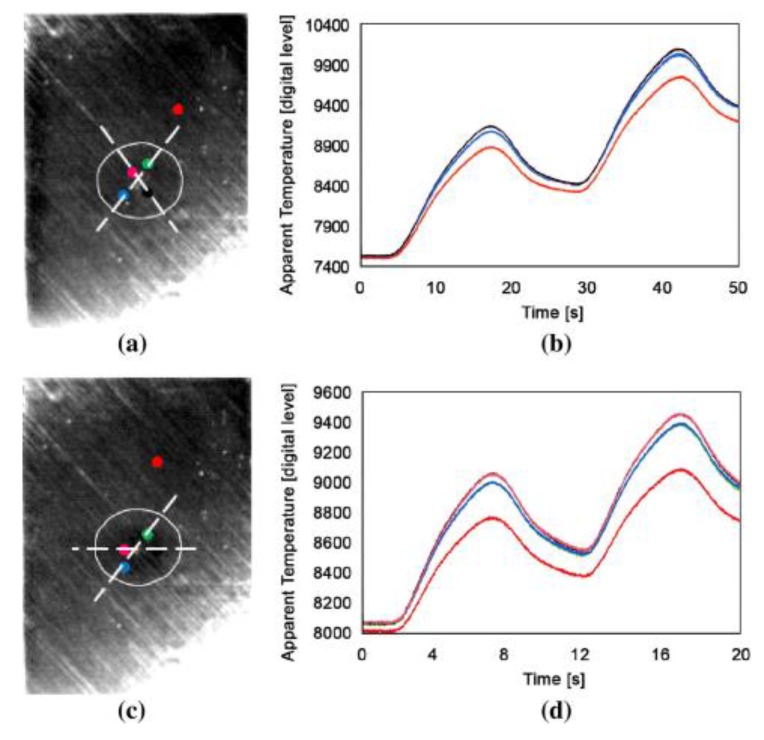
Ultrasonic lock-in thermography results on a composite panel with BVID, with permission from. Figure (**a**,**c**) shows the lock-in results with delamination at −45° and +/−45°, and −45° and 90° respectively, whilst figure (**b**,**d**) illustrates the apparent temperature results at 0.04 Hz and 0.1 Hz, respectively. [110].

Specifically for the aerospace industry, UST is mainly used for the inspection of micro-cracks (of the order of microns) in aluminium aerospace components [102], aircraft engine fan blades [103,104] and composite primary and secondary structures. Early testing on composite materials included the evaluation of thick multilayer carbon/carbon composites used in wing leading edges of the space shuttle [105], the inspection of F16 airplane main landing gears [106] and the detection of internal delaminations in graphite-fibre composites and disbonds in airplane vertical stabilizers [107]. Unlike OST, the “clapping” motion and friction (“rubbing”) of closed cracks stimulated by ultrasonic waves is an “ideal” condition for UST. Renshaw et al. [108] have shown that heat generation in damage regions during a thermosonics experiment is mainly due to three different mechanisms: (i) frictional rubbing of contact regions of damage interfaces; (ii) plastic deformations during crack or damage growth that generate heat in the plastic zone surrounding these areas; and (iii) viscoelastic losses that generate significant heat in regions of stress concentration (i.e., around delaminations and other defects). Polimeno et al. [99] developed a compact thermosonics inspection system with a microbolometer array camera to detect artificial delamination (i.e., an embedded Teflon patch) in composite materials. The authors used a parameter, known as Heating Index [109], to predict the vibration level in the presence of vibrations governed by “acoustic chaos”. The calculation of the Heating Index involved calibration methods and accurate measurements of the strain energy for each test case. Gaudenzi et al. [110] compared the performance of UST with standard OST and ultrasonic phased array system on a CFRP samples undergone to low velocity impact damage. The authors found that UST provided a quick and reliable estimation of material micro-defects. Figure 13 shows the experimental UST results of a composite sample with delaminations obtained at an impact energy of 20 J.

**Figure 14:**
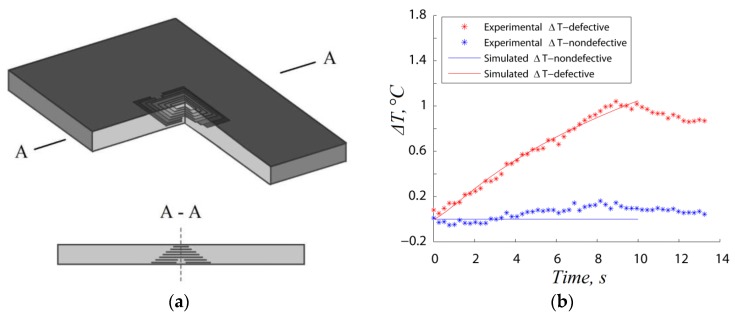
Modelling of impact damage as a pyramid-like defect shape (**a**) and the comparison between experimental and numerical temperature results (**b**), from [114].

Vavilov et al. [111] developed an analytical model for ultrasonic stimulated thermography and compared the analytical results with experimental data on a carbon-carbon composite sample with BVID. Following these experimental investigations, numerical models were also developed to simulate UST and study qualitatively the crack heating phenomenon at damage location caused by internal and external energy losses [112,113]. Derusova et al. [114] numerically modelled with finite element (FE) the impact damage in a carbon epoxy composite as a pyramid-like defect with a set of delaminations acting as air-filled heat sources. As illustrated in Figure 14, the authors found a good agreement between the experimental temperature variations in a defective area and the numerical results. They also were able to estimate the energy generated by each defect (i.e., the “defect power”). 

Parvasi et al. [115] used a coupled thermo-electro-mechanical FE model to simulate the interaction of ultrasonic wave transmitted by low-power piezoceramic-based transducer with the material damage and the associated temperature response. The authors used a static-kinetic exponential decay model to simulate the frictional heating at crack interfaces. However, the exposure to high-power excitation generated by the acoustic/ultrasonic horn used in UST may even further degrade the structural integrity of the components [116]. Zalameda et al. [117] developed a contactless ultrasonic stimulated thermography with air-coupled transducers for damage detection of an helicopter blade sandwich. The acoustic source consisted of an array of four amplified loudspeakers emitting pulses at different frequencies ranging between 700 Hz and 1.3 kHz. The authors compared their results with standard flash thermography and were able to reveal the presence of disbonds between the skin and core.

### 3.1. Ultrasonic Stimulated Thermography Using the Local Damage Resonance Effect

The ultrasonic horn used in thermosonics is a bulky and crude means of exciting high-power vibrations. The coupling between the test specimen and the horn typically results in an uncontrolled generation of frequency components known as “acoustic chaos” [99]. Such a condition makes UST “non-reproducible”, thus leading to cracks being undetected if sufficient vibrational energy is not applied at the crack location. To overcome this issue, a new material elastic effect, known as local resonance defect (LDR), has recently gained considerable attention as it allows selective ultrasonic activation and higher sensitivity to the presence of structural flaws [118,119,120]. LDR shall be referred as the interaction of acoustic/ultrasonic waves with the damaged area at a frequency matching the defect resonance, which results in a substantial enhancement of the vibration amplitude only in the localised damaged region [121]. Assuming that the internal structural flaw such as a delamination in a composite laminate of thickness *h* is represented by a flat bottom hole, i.e., a thin circular defect of radius *r*, the expression of the LDR frequency *f_d_* becomes [119]:

(13) $$f_{d}≅\frac{1.6d}{r^{2}}\sqrt{\frac{E}{12\rho \left(1-\upsilon^{2}\right)}}$$

where *E* and *υ* are the effective elastic modulus and Poisson’s ratio of the composite laminate, respectively, *ρ* is the density and *d* is the depth of the portion of volume below the defect. Equation (13) corresponds to the first bending mode of a circular plate with clamped boundaries. For a quadratic-shaped defect with side length *l*, Equation (13) becomes [121]:

(14) $$f_{d}≅\frac{4\pi d}{3l^{2}}\sqrt{\frac{E}{6\rho \left(1-\upsilon^{2}\right)}}$$

**Figure 15:**
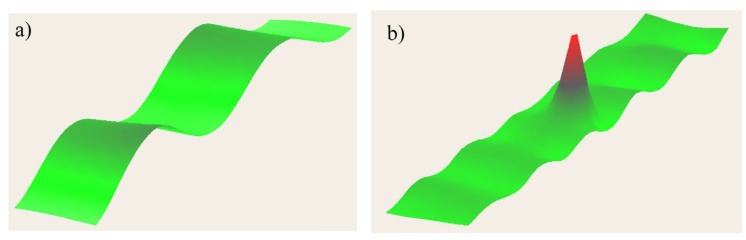
Vibration patterns at the GFRP specimen at 3.4 kHz (**a**) and at the LDR frequency, 20.9 kHz, on a GFRP sample (**b**), with permission from [119].

In the case of a notch of width *w*, the LDR frequency is [119]:

(15) $$f_{d}≅\frac{2\pi }{w^{2}}\sqrt{\frac{B_{s}}{3\rho d}}$$

where *B_s_* is the bending stiffness given by $B_{s}=Ed^{3}/12\left(1-\nu^{2}\right)$. Figure 15 shows an example of LDR frequency (3.4 kHz) on a glass fibre reinforced plastic (GFRP) sample.

### 3.2. Nonlinear Ultrasonic Stimulated Thermography 

**Figure 16:**
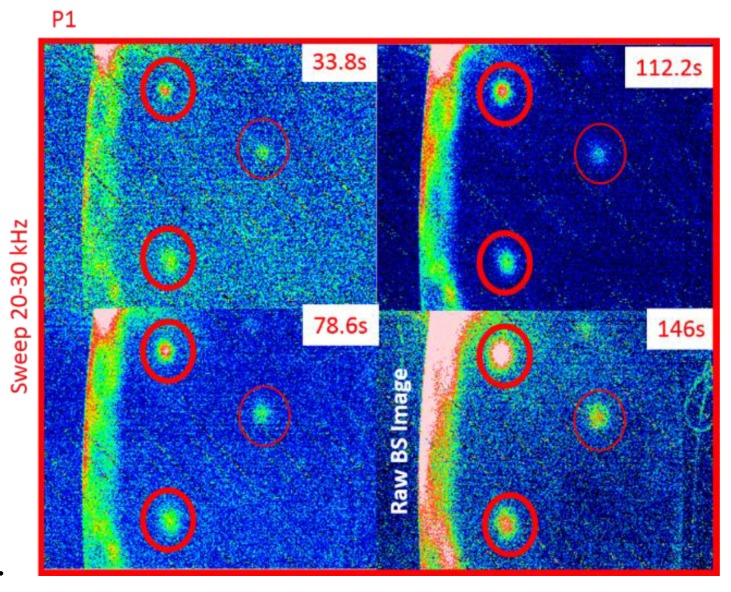
Nonlinear ultrasonic stimulated results thermography on a composite stiffened panel while conducting a sweep between 20 kHz and 30 kHz, with permission from [28].

LDR also exhibits transitions from linear to nonlinear regime with the effect of generating higher and sub-harmonics of the input frequency [119,120]. Such an effect is typically known as “nonlinear LDR” [121], and the combination of thermosonics with this effect is known as “Nonlinear Ultrasonic Stimulated Thermography” (NUST). Solodov et al. [122] used NUST to improve the driving frequency-selection process for the generation of frictional heat on a composite sample using commercial loudspeakers. They matched the NUST thermal results with those obtained using a shearography system. Fierro et al. developed a nonlinear narrow sweep excitation method for NUST in order to detect delamination fatigue damage on aluminium sample [123] and debonds on an aerospace composite stiffener panel (see Figure 16) [28]. In the last paper, the authors developed a coupled thermal-structural finite element model to analyse the generation of frictional heat and the associated nonlinear elastic effects on the composite sample. Good agreement between the numerical and experimental results was found. Recently, Dionysopoulos et al. [124] used NUST with frequency modulated signals generated by the crack in order to image barely visible impact damage on an aerospace composite panel.

## 4. Eddy Current Stimulated Thermography

**Figure 17:**
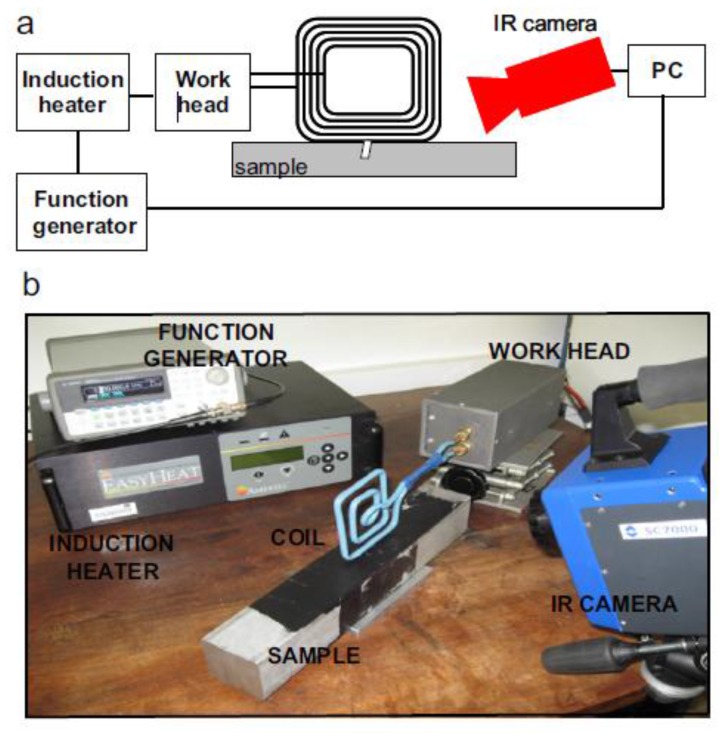
System set-up diagram (**a**) and experimental set-up (**b**) of pulsed ECST, with permission from [125].

Eddy Current Stimulated Thermography (ECST) induces eddy currents in a conductive material via a coil and the associated induction heating is measured with an IR camera [125]. When eddy currents encounter a material damage (discontinuity), they are forced to divert and change the direction of the current flow. This leads to areas of increased and decreased eddy current density resulting in relatively hot and cool areas due to Joule heating. Typically, a high-current eddy current pulse with typical central frequency ranging between 150 and 450 kHz is used for excitation (also known as “pulsed ECST”). The excitation period can change from few milliseconds for high-conductivity materials (e.g., metals) to few seconds for low-conductivity specimens (e.g., plastics and carbon fibre reinforced plastic laminates). Figure 17 shows the experimental set-up for pulsed ECST.

Because of its working principle, in pulsed ECST, the application of heat is not limited to the sample surface, like in optical pulsed thermography, and it can reach a certain depth. For a homogeneous field excitation parallel to the surface, the penetration depth of a magnetic field in a material is governed by the skin effect. The skin depth or penetration depth, *δ* is given by [126]:

(16) $$\delta =\frac{1}{\sqrt{f\pi m_{p}\sigma }}$$

where *f* is the frequency of the pulse excitation and *m_p_* and *σ* are the magnetic permeability (H/m) and electrical conductivity of the material (S/m), respectively. Typical electrical conductivity of unidirectional single layered CFRP is approximately 5 MS/m in the longitudinal direction and 1 kS/m in the transversal one [127]. Moreover, inductors used for ECST of composites operate at high excitation frequencies from 100 kHz to 100 MHz [128]. Hence, the skin depth for CFRP with small conductivity (~1 kS/m) and no magnetic permeability is significantly great (~50 mm under 100 kHz excitation) [129].

According to [54] the Green’s function formulation, the estimation of the temperature profile in ECST for an infinite length and finite thickness plate can be expressed via the following two equations, for the heating phase ([29,130]):

(17) $$T\left(0,t\right)=\frac{Q2\sqrt{t}}{\rho c\sqrt{\pi \alpha }}\propto a_{r}\sqrt{\frac{t}{t_{p}}}\mathrm{for}\;t<t_{p}$$

and the cooling phase:

(18) $$T\left(0,t\right)=\frac{Q}{\rho cL}\left\{1+2\sum_{n=1}^{\infty }\exp\left[-\frac{n^{2}\pi^{2}}{L^{2}}\alpha \left(t-t_{p}\right)\right]\right\}\propto \frac{1}{\sqrt{a_{n}\left(t-t_{p}\right)}}\;\mathrm{for}\;t<t_{p}$$

where $Q={\left|J_{s}\right|}^{2}t/\sigma$ is the generated resistive heat with *J_s_* the eddy current density, *t_p_* is the duration of the heating pulse and *L* is the thickness of the sample. The coefficient *a_r_* is the amplitude of temperature rise determined by local electric conductivity variations at the damage location, whilst *a_n_* is the normalized temperature decay rate, determined by local thermal property changes and material defect dimensions. According to the theory of electromagnetic induction, some power is lost in the coil and cannot be used for excitation. Hence, the heating efficiency *η_c_* can be estimated as follows [131]:

(19) $$\eta_{c}\approx {\left(1+\frac{2h_{c}}{r_{c}}\sqrt{\frac{\sigma \mu_{pc}}{\mu_{p}\sigma_{c}}}\right)}^{-1}$$

where *h_c_* is the distance of the coil form the material surface (also known as “lift-off distance”), *r_c_* is the radius of the coil, *μ_pc_* and *σ_c_* are the magnetic permeability and electrical conductivity of the coil, respectively. Equation (19) shows that to increase the heat efficiency, the permeability of the coil should be as close as possible to 1 and the electrical conductivity of the coil should be as high as possible. Typical values of *h_c_* and *r_c_* are 2–10 mm and 2–2.5 mm, respectively.

Equation (19) is generally applicable to a wide range of materials and material failure scenarios for aerospace applications, including corrosion on aluminium samples [132,133] and crack/delamination in carbon fibre composites [134]. This last material has complex electromagnetic properties in which electrically conductive carbon fibres are embedded into a dielectric resin matrix. Since the electrical and thermal conductivity are the greatest along the fibre direction, recent advances of ECST involve the determination of buried defects in the dielectric phase of composite materials (resin), such as moisture ingress, aging of the material due to service or environmental/thermal exposure, voids and delamination [134]. 

**Figure 18:**
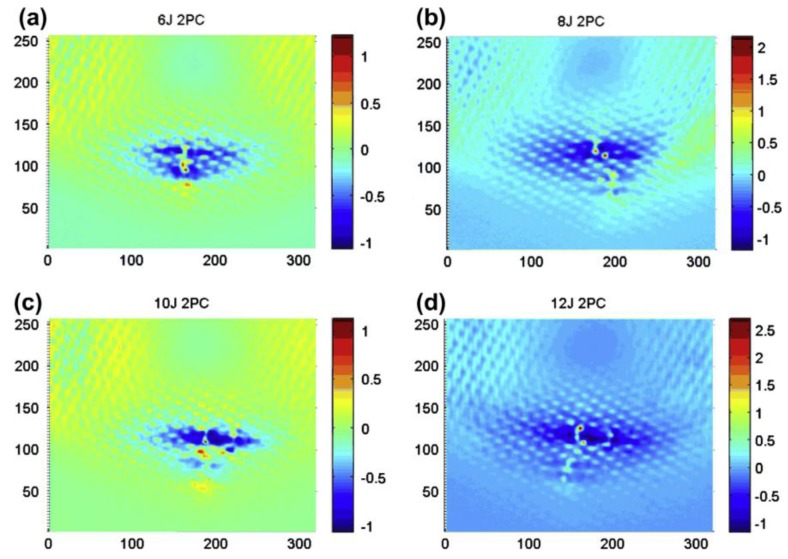
ECST results combined with PCT on a CFRP composite sample impacted at 6 J (**a**); 8 J (**b**); 10 J (**c**) and 12 J (**d**), with permission from [135].

He et al. [135] and Liang et al. [136] used the ECST combined with the principal components thermography (PCT) to detect material damage on CFRP samples impacted with different energies between 4 J and 12 J (Figure 18).

**Figure 19:**
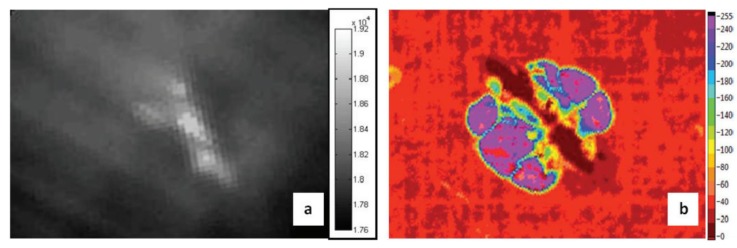
Comparison between eddy current stimulated thermography (**a**) and ultrasonic C-Scan (**b**) on impact damaged CFRP sample, with permission from [139].

Ishikawa et al. [137] and He and Yang [138] converted thermal ECST data from time domain to frequency domain and used phase images in order to increase the detectability of delamination in CFRP sample. Since the influence of non-uniform heating is periodically suppressed in phase images, the authors found that the damage imaging resolution (accuracy) was enhanced. Renil Thomas and Balasubramaniam [139] developed a scanning induction method for ECST on impact damaged CFRP samples, in which the induction coil moved over the sample at the speed of ~40 mm/s and the temperature profiles caused by Joule heating were captured with an IR camera. The final ECST image was compared with a standard ultrasonic C-Scan showing good agreement in terms of damage location and severity (Figure 19).

Yang and He [129] combined a selective heating thermography through electromagnetic induction with cross correlation match filtering for the inspection of damaged CFRP composites. This technique reduced the non-uniform heating and lateral blurring effects that are typical of ECST. 

ECST can be easily automated/robotised and has shown high sensitivity for damage quantification on dry, wet and consolidated CFRP. Moreover, since eddy currents flow parallel to the surface, it is very complex to detect delamination in CFRP unless there are interlaminar fibre contacts. To overcome this issue, an alternative technique recently developed for aerospace components is the Capacitive Imaging (CI) method [140], which is based on the variation of dielectric properties of the resin matrix in order to enable detection of delamination caused by impact damage. Further studies of CI combined to thermography are under development to verify its effectiveness for aerospace applications. 

### 4.1. Microwave Thermography

Microwave Thermography (MWT) uses electromagnetic radiation with frequencies ranging between 300 MHz and 30 GHz (microwaves) in order to generate heat due to the dielectric loss of materials [141]. These frequencies are chosen by international agreement in order to minimise interference with aircarft communication services. The idea behind MWT is that where there is a damage such as delaminations, cracks and voids, the water trapped in the open defect is released for vapour tension and the associated heat is measured by the IR camera. In MWT, the dissipated power per unit volume *P* in dielectric media is provided by the following equation [19,142]:

(20) $$P=2\pi f\varepsilon_{0}\varepsilon^{\prime \prime }E_{f}^{2}$$

where *f* is the frequency of the microwave excitation, *E_f_* is the electric field, *ε*_0_ is the permittivity of the air and $\varepsilon^{\prime \prime }$ is the loss factor that quantifies the power dissipation. The temperature change at heating time *t* by power dissipation of continuous microwave is [142]:

(21) $$T\left(t\right)=\frac{P}{\rho c}$$

From Equation (21), it can be seen that the temperature rise is approximately linear with time. The main advantage of the MWT is its volumetric character, meaning that a certain volume of the specimen can be heated at once, thus leading to a very fast inspection of large parts. However, according to Equations (20) and (21), the heating ratio is dependent not only on the thermal properties of the material, but also on its electrical properties. MWT has been used only recently for the inspection of CFRP composites for aerospace applications [143]. Palumbo et al. [144] compared MWT with LIT for the detection of impact damage on sandwich composites. The authors provided a quantitative analysis of the damaged area by using a CT-Scan (X-ray tomography) and showed that both LIT and MWT were able to provide similar results, whit the additional advantage of the latter technique being much faster (only two seconds of heating and simple signal processing). 

## 5. Material-Based Thermography

One of the key parameters in IRT is the selection of the thermal stimulation source. This selection determines the physical constraints of the thermographic system such as: (i) the requirements of clearance and accessibility of the component under examination; (ii) the power consumption in order to comply with other aircraft/spacecraft systems; (iii) the inspection costs and (iv) the limitations in terms of resolution of the analysis and its effectiveness in identifying in-depth defects. During the last two decades, beside optical thermal stimulation, ultrasonic excitation and electromagnetic radiation, several alternative stimulation sources have been investigated to enhance the resolution of the thermal analysis while reducing the power requirement [19]. In particular, by analysing the results obtained with all these different excitation techniques, it is possible to identify three major drawbacks linked with the use of external thermal stimulation. Firstly, the need of uniform thermal heating involves accurate position of the external heat source, which, inevitably, limits in-situ analysis. Secondly, there is a need for cost-effective thermal excitation systems that do not require high input power. Thirdly, thermal responses in fibre composite laminates, due to their anisotropic structure behaviour, are generally affected by a high level of noise that needs to be filtered with advanced signal processing techniques in order to avoid misdetection of damaged areas or false positives. 

Based on these considerations, a number of authors has investigated alternative strategies to current external thermal excitation sources in composites. In this section, we identify two different approaches, the “direct material-based thermography” (DMT) and the “indirect material-based thermography” (IMT). The DMT method consists of exploiting the specific properties of the material under analysis in order to assess its internal health status. This can be done in conductive metal parts and composite laminates by using one of the components (e.g., the resin matrix or the reinforcement fibre) to generate heat via Joule effect. The IMT approach, instead, involves the embodiment of additional thermoresistive components within the composite laminate during the manufacturing process in order to generate internal thermal stimulation. In both approaches, the material itself is able to autonomously assess its internal health state without the use of external excitation heaters or flash lamps and, similarly to traditional IRT, the heat flow interacting with the material damage can be detected with an IR camera.

### 5.1. Direct Material-Based Thermography

**Figure 20:**
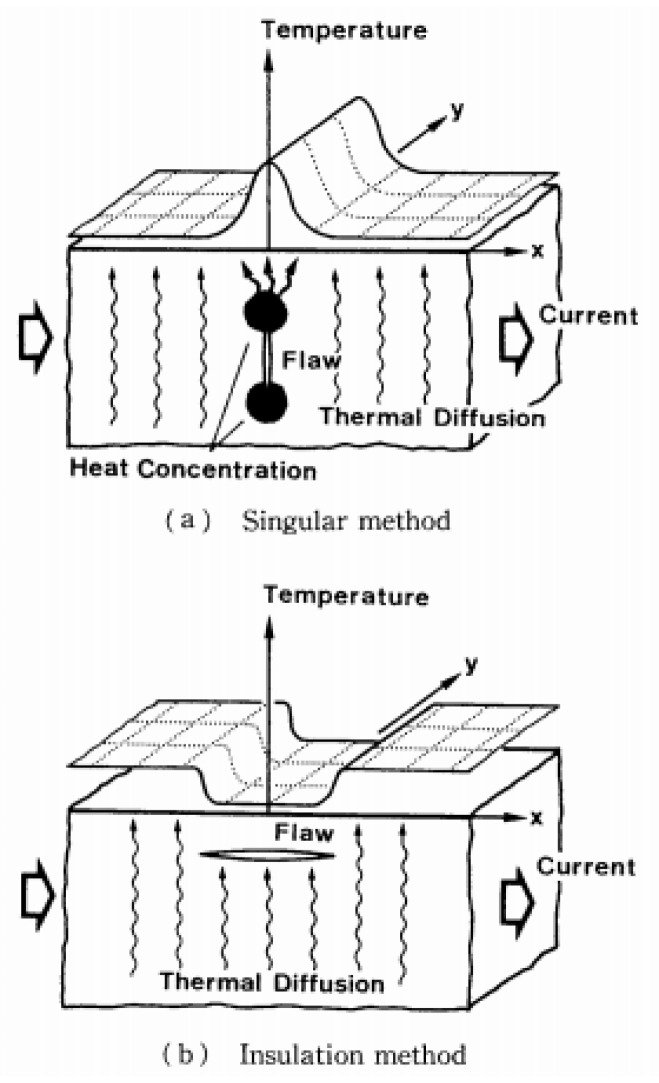
Illustration of the “singular method” (**a**) and the “insulation method” (**b**), with permission from [31].

Sagami et al. [31] developed two different DMT techniques based on the application of electrical current on electrically conductive samples and the associated generation of heat via Joule effect. The first technique, called “singular method” (see Figure 20a), is based on the increase of the current density near the tip of a intralaminar crack (oriented at 90°) in a stainless steel plate as result of the interaction with the stream line of an applied DC current. This increase, in turn, results in a raise of the temperature field with a significant heat concentration at the crack tip that can be easily detected from the top surface with an IR camera. Further experiments with cracks at 0°, 30°, 45° and 60° were carried out by observing a less significant heat concentration with the reduction of the inclination angle due to the decreasing behaviour of the current intensity factor. The “singular method” depends on the intensity of the applied current field and on the thermal diffusion rate. Indeed, high values of thermal conductivity may lead to poor detection of flaws when the analysis is not conducted in a short inspection time. 

The second approach, named “insulation method” (see Figure 20b), was designed for the detection of interlaminar flaws in CFRP composites. In this case, the crack tip does not generate any singularities in the temperature field but the defect acts as an insulator to the thermal conduction, thus resulting in a low-temperature region detectable from the top surface. This technique was experimentally validated by applying a DC current to a twelve layers CFRP sample (500 × 100 mm) with an internal patch of polyimide film. The electrical connections were obtained by removing the resin matrix from two 100 × 100 mm areas on the two edges. Experimental results showed that both DMT methods are able to detect the presence of internal flaws. However, high input currents (i.e., ~20 A for the “singular method” and ~40 A for the “insulation method”) are needed.

#### 5.1.1. Electrical Resistance Change Method and Thermography

The idea of exploiting the conductive properties of carbon fibres in CFRP composites for the autonomous assessment of their internal health status has been investigated in the “electrical resistance change method” (ERCM) [145,146,147]. In this technique, carbon fibres are used as “sensors” exploiting their electrical conductivity and variations in the electrical resistance are monitored to detect cracks or indentation damage. Although ERCM was proved to be efficient in detecting early stage material flaws, its reliability is not high enough for practical applications. This is mainly caused by: (i) fluctuations of the electrical conductivity of laminated composites; (ii) the nature of electrical connections that require a large amount of wiring for complex structures and have strong dependence with temperature and (iii) the limitation of ERCM for damage detection in thick laminates. 

**Figure 21:**
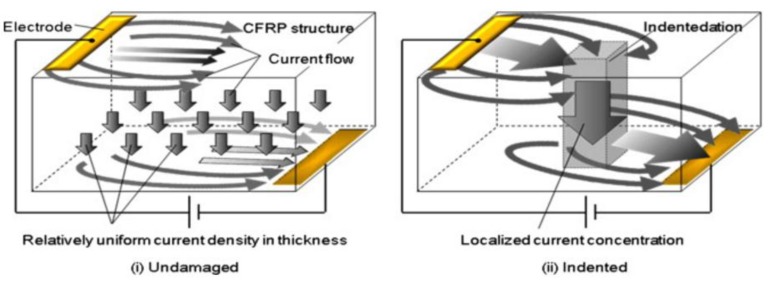
Schematic of the technique developed by Suzuki et al.: difference in the path of the electrical current in the case of undamaged and indented areas, with permission from [148].

Suzuki et al. [148] recently combined ERCM with thermography in order to detect damage in aerospace components. An electrical voltage was applied on the two outermost layers of a composite laminate and the variations of the temperature field were observed with an IR camera. Since indentation damage increases the contact between the fibres of two separate layers and reduces the matrix interphase, the intralaminar electrical resistivity in the damage area decreases, thus leading to a local increase of the current density that can be identified on the material’s surface as a hot spot (Figure 21).

The authors evaluated the electrical properties of an IM600/133 CFRP composite laminate both experimentally and numerically to test the effectiveness of the proposed ERCM combined to thermography. Particularly, undamaged, indented and delaminated CFRP samples were investigated using an input voltage ranging between 5 and 10 V applied on two electrodes. Results showed that delamination of 0.15 mm could be easily detected by applying 5 V, thus generating a temperature variation of more than 30 °C between damaged and undamaged areas. As for internal defects, the technique was not able to detect the presence of an internal delamination, showing only a slight decrease of the top surface temperature in correspondence with the damaged area. An advantage of this method is that traditional aircraft lightening protection strips can be used as electrical contacts and wiring, thus simplifying the manufacturing procedure and reducing production costs, as demonstrated in a further work by the same authors [149]. 

Grammatikos et al. [150] also combined ERCM with thermography and compared this method with different optical thermographic techniques including PT, PPT and LIT in order to monitor the structural integrity of aircraft composite parts. The internal heat stimulus was provided by a square electric pulse at low frequency applied to the bulk laminate in order generate a diffuse thermal field via Joule heating. The authors assumed that if an undamaged carbon layer is characterised by a resistance *R*_1_, a damaged area will be characterised by a higher resistance *R*_1_ = *nR*_2_ (with *n* > 1) due to the presence of the internal flaw (e.g., crack, delamination or BVID). By considering the 1st law of thermodynamics and both the Ohm’s and Joule’s law, the electrical power *P*_1_ and *P*_2_ for both an undamaged and damaged composite can be expressed as follows:

(22) $$P_{1}=I^{2}R_{1}\;\mathrm{and}\;P_{2}=I^{2}R_{2}=I^{2}R_{1}n$$

with *I* the input current. Assuming that *I* is a constant, the increase in the electrical resistance in the damaged area will lead to an increase of the electric power since *P*_1_ = *nP*_2_ , which translates in a temperature increase at damage location. To validate this finding, experimental data were collected by testing CFRP square coupons 60 × 60 × 1 mm and electrical connections were obtained by removing resin from both the edges and applying silver paint and silver loaded adhesive tape, showing good results in terms of accuracy. 

In addition, since thermal waves propagate through the thickness of the sample, the thermal-electrical properties along the through-thickness direction constitute a key parameter for the effectiveness of ERCM and thermography. In particular, the electrical conductivity along the sample’s thickness is given by the random interlaminar fibre-fibre connections that lead to a pattern of locations with higher conductivity. This, in turn, determines the anisotropy of the heat flow propagation through the laminate. As a consequence, in order to increase the number of random contacts and enhance the heating front propagation, 0.5% *w*/*w* of carbon nanotubes (CNTs) was included within the epoxy matrix [151]. The nanomodified samples were subjected to low velocity impacts at different energies (3 and 4 J) and the material damage was evaluated in “live mode” by first recording the surface temperature during the application of electrical current at 10 A for 60 s and then during the cooling down process for other 150 s. Results from these tests showed that the difference in the temperature field between damaged and undamaged areas was higher during the heating phase rather than the cooling one. The authors explained these results with the different mechanisms of the two processes: whilst during heating the heat flow is directed through the injection path, during the cooling phase part of the thermal energy is dissipated through the edges of the monitored surface. However, these observations seem to be in contrast with what is usually seen in traditional thermography with external heat sources as cooling ramps are more efficient in detecting internal flaws [152]. In order to enhance the resolution of the results achieved in “live mode” and, simultaneously, keep the power consumption to lower levels, PPT was employed by connecting the camera with a pulse generator and applying 200 mA (0.5 V) to the sample for 50 s. The comparison between the PPT phase images and traditional C-scans showed that in case of CNT-modified samples, ERCM combined to thermography was able to reveal the presence of low velocity impacts through the identification of cold-spots on the surface. 

### 5.2. Indirect Material-Based Thermography

Although DMT has demonstrated its effectiveness as material enabled NDE technique, electrical connections applied directly to fibres may lead to thermomechanical stresses especially on the edges of the composite sample if the power required for the inspection or the time of the analysis are very high. In addition, DMT only works if the material is characterised by good electro-thermal properties, thus ruling out completely any glass fibre composite application. IMT is, conversely, based on a different approach than DMT, in which a thermoresistive phase is added within the material structure and acts as an embedded heat source that can be used for both structural and non-structural purposes [34].

#### 5.2.1. Metal-Based Thermography

Ahmed et al. [32] developed a technique called “Heat Emitting Layer” (HEL) for Metal-based Thermography (MT), which was based on the development of an engineered additional layer fully integrated into the laminate structure in order to guarantee a fast inspection of large areas. The HEL was manufactured using AISI316 type stainless steel wires with a diameter of 70 μm woven into a satin 8H glass fabric at specific intervals and was then used within the lamination sequence of several GFRP laminates in which polyamide (PI) foils where embedded to simulate the presence of internal defects (Figure 21).

**Figure 22:**
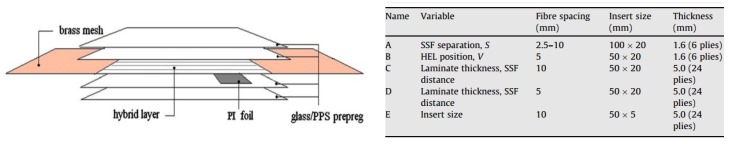
HEL laminates: Schematic lay-up of the HEL laminates and specifications of the different laminates prepared during the experimental campaign, with permission from [32].

As it is possible to see from Figure 22, the experimental campaign was aimed to investigate several aspects of MT such as the inter-distance between stainless steel wires within the HEL layer, its position along the laminate’s thickness and the resolution of the technique with increasing distance between the thermal emitting layer and the embedded defect. Upon an accurate analysis of MT thermal results, the authors conclude that the fibre spacing played an important role in the resolution of the thermogram. Indeed, by decreasing the inter-wire distance from 10 to 2.5 mm, the inspection produced a more defined view of the PI insert due to the higher contrast between wires and the damaged area. In addition, MT experimental results and numerical data collected from finite element analysis showed that by placing the HEL on the bottom of the laminate, the propagation of the thermal front through the thickness was more uniform, thus guaranteeing the highest resolution. However, because of the distance from the top surface, the difference in temperature between damaged and undamaged areas was only 0.5 °C, which means that high injection currents were required. In particular, when low power was used on samples with different thickness (~0.02 W/cm^2^ for the 6 plies laminate and ~0.13 W/cm^2^ for the 24 plies one) not all the inserts could be detected even when the excitation current was applied for 10 s. By increasing the input power to ~0.19 W/cm^2^, most of defects became visible for the 6 plies laminates and two out of three patches were detectable for the 24 layers one (~0.42 W/cm^2^). It is important to highlight that when MT is applied on structures more complex than plates, other parameters such as the waviness degree of the stainless steel wires or the reflective surface of curved parts become fundamental for the correct detection of flaws and need to be taken in account in order to enhance the effectiveness of this technique. 

Following a similar approach, Orlowska et al. [153] proposed a further development of MT by embedding a specially designed 3D electrical grid composed of through-the-layer (vertical) and surface-layer (horizontal) metal elements. The grid was designed so that its mechanical properties where the same as the composite material and, in case of loss of adhesion between two layers in a localised portion of the structure, the embedded grid could fracture simultaneously and break the conductive mesh. This local disruption of the electrical field generates a decrease in the thermal density at damage location that can be experimentally observed by a long-wave IR camera. Numerical finite element analysis was also conducted by considering different thicknesses of the sample and various dimensions of the internal delamination (represented by an embedded Teflon patch). Results indicated that the temperature difference between a damaged and undamaged area was strongly affected by the position along the thickness of the delamination, thus showing a temperature difference of only 0.2 °C in the case of a patch inserted within a 24 layers sample. The extension of the patch also represented a key parameter for MT and could potentially constitute a limitation by showing only 0.1 °C of temperature difference in case of a 6 × 6 mm embedded patch. By using a non-stationary thermal excitation with a rectangular pulse of 20 s, temperature differences were enhanced up to 3.63 °C. This method was validated experimentally by manufacturing a 8 layers GFRP laminate with a grid of Aluchrome wires with 120 μm of diameter. In order to embed the conductive mesh within the laminate, tiny holes were drilled within the thickness of the sample and the resistive elements were placed in three parallel lines. One of the connection was left intentionally detached in order to simulate the disruption of the conductive grid generated by an internal delamination and a current of 1.1 A was applied to the entire structure. Results from the thermal image demonstrated a good accuracy of the method in terms of damage detection. However, as the authors acknowledged in the paper [153], in order to avoid the weakening of the material due to the drilling operation, further work is still required in order to design a different manufacturing procedure in which the conductor wires are knitted on the different fibre layers before the impregnation with the resin. 

#### 5.2.2. Carbon Nanotubes-Based Thermography

**Figure 23:**
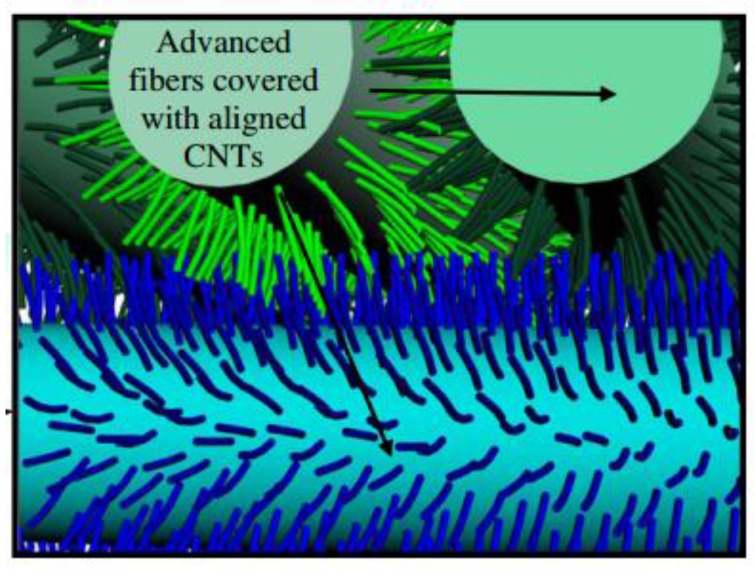
CNT/Alumina hybrid composite: internal nanoscale interaction between alumina fibres and carbon nanotubes, with permission from [154].

The simultaneity of damage in both the embedded sensing device/mesh/grid and the sample itself may limit the sensitive of MT, as false positives could be identified in case of damaged sensor and no structural damage. To overcome this limitation, one of the most promising approaches is to intend the material-enabled thermography feature as one of the multiple functions of a more complex multi-physics material system. In other words, the additional phase within the laminate’s structure needs to be fully integrated with the existent phases in order to work not only as a sensing device but also as a combined structural reinforcement, thus leading to a multifunctional material system whose structural and non-structural properties results enhanced in comparison with a traditional laminate. As seen in Section 5.1.1, the inclusion of CNTs within a resin matrix enhances the thermomechanical properties and, in turn, increases the resolution of carbon nanotubes-based thermography (CNTT) when a current is applied at the edges of a CFRP laminate. By following a similar approach, de Villoria et al. [33] developed a CNTT technique termed by the authors “Nano-Engineered Thermal NDE” (NET-NDE) by manufacturing hybrid alumina fabrics with aligned CNTs forest. The 30 μm multi-walled nanotubes were grown through chemical vapour deposition on the surface of the satin cloth following a “Mohawk” morphology and were characterised by a diameter of 17 nm (Figure 23). Composite specimens were manufactured via hand lay-up impregnating three layers of fabric with epoxy matrix. The final laminates were characterised by 50 vol % of alumina fibres and 2 vol % of CNTs. The presence of the nano-scale phase within the composite was able to bridge the ply interfaces, thus enhancing both intralaminar and interlaminar strength, as demonstrated by previous works from the same research group [154,155]. In order to test the efficiency of CNTT, an in-situ thermal analysis was performed by applying a 6.8 V (2 W) on the edges of an hybrid sample with a 6.5 mm hole subject to a failure tensile test. Results showed that superficial cracks opening and stress concentration areas could be easily followed via thermographic inspection by monitoring the variation of the thermal gradient on the damaged areas.

Good results were also observed from tests conducted on a riveted panel with internal cracks not visible from the top surface using input voltages of 0.16 V and 12.V, and on damaged composite plate undergo to low velocity impact by applying 0.34 V on both ends.

#### 5.2.3. Shape Memory Alloys-Based Rapid Thermography

In order to develop a fully multifunctional material based on carbon composites that can be used for aerospace applications, another approach is based on the development of a new hybrid material that includes Shape Memory Alloy (SMA) wires within the lamination sequence of a traditional CFRP parts. CFRP hybridisation with SMAs has been investigated by several authors in order to exploit the unique properties of superelasticity and shape memory effect of SMAs to increase impact resistance and reduce the extent of internal delamination [156,157]. These properties rise from the transitions between two different crystalline structures (martensite and austenite) that can be activated by applying temperature gradients or by loading the material with an external force. By combining the enhancement of the mechanical properties with strain sensing, damage localisation and de-icing features, Pinto et al. [34] developed a new multifunctional material embedding SMA wires within traditional CFRP composites. This technique, named Shape Memory Alloys-based rapid Thermography (SMArT), was based on the generation of an in situ low-power resisting heating that allowed the detection and the assessment of structural damage in aerospace composite structures. Experiments to validate SMArT were undertaken on several samples in order to analyse different variables such as dimensions of the embedded damage (20 × 20 mm, 10 × 10 mm and 5 × 5 mm), resolution of the analysis, effect of the feeding current intensity (0.1–0.9 A) and relative position of the wires within the sample’s thickness. Results from the experimental campaign showed that the presence of the embedded hybrid phase was able to guarantee uniform heating flow through the sample’s thickness. When thermal waves propagating from the SMA interact with the internal defect, they are slowed down due to the different thermal diffusion rate and generate a localised variation in the apparent temperature on the sample’s surface that can be detected with an IR camera. 

**Figure 24:**
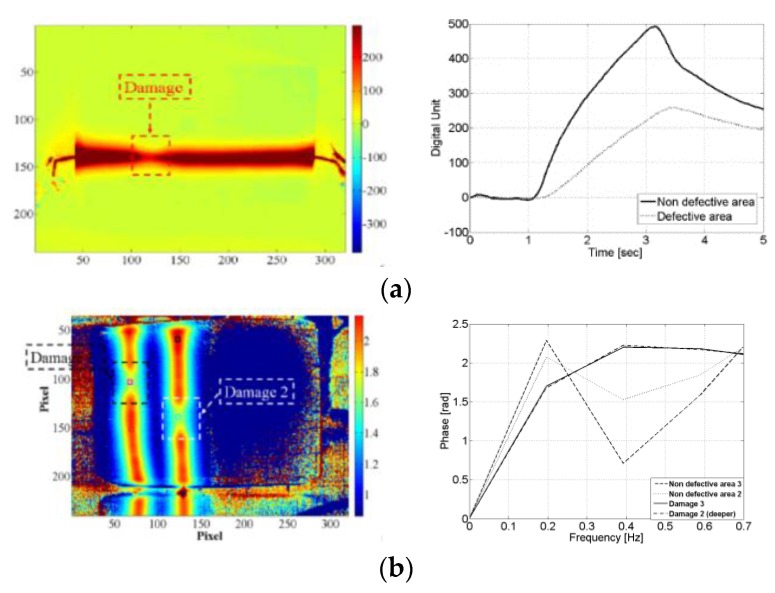
SMArt multifunctional laminate: time history of the temperature difference variation between damaged and undamaged part (4 plies CFRP in cross-ply stacking sequence, single damage) (**a**); phase profile of two different damage sites (16 plies, cross-ply stacking sequence, multi-damage) (**b**), with permission from [34].

From a mathematical point of view, after a uniform impulse of thermal energy is released instantaneously by the SMA wire at time *t* = 0 and propagates along the through-the-thickness direction, the 1D analytical model of the temperature rise *T_nd_*(*h,t*) on the surface of an undamaged material is given by [158]:

(23) $$T_{nd}\left(h,t\right)=\frac{Q_{0}}{4\pi kt}\left[\sum_{m=0}^{\infty }e^{-\frac{{\left(2mL+h\right)}^{2}}{4\alpha t}}+\sum_{m=0}^{\infty }e^{-\frac{{\left[2\left(m+1\right)L-h\right]}^{2}}{4\alpha t}}\right]$$

where *h* is depth location of the wire and *L* is the thickness of the composite plate. Assuming a delamination-like defect layer of thickness *d* lying beneath the material surface in a plane parallel to the surface, the temperature rise *T_d_*(*h,t*) on the surface of the defective material becomes:

(24) $$T_{d}\left(h,t\right)=\frac{Q_{0}}{4\pi kt}\left[\sum_{m=0}^{\infty }{\left(1-\Gamma \right)}^{2m+1}\sum_{n=0}^{\infty }\Gamma^{n}e^{-\frac{{\left(2mL+h+2nd\right)}^{2}}{4\alpha t}}+\sum_{m=0}^{\infty }{\left(1-\Gamma \right)}^{2m+1}\sum_{n=0}^{\infty }\Gamma^{n}e^{-\frac{{\left[2\left(m+1\right)L-h+2nd\right]}^{2}}{4\alpha t}}\right]$$

where Γ is the effective thermal reflectivity of the defect. For a circular defect of diameter *D*, the diffusion distance is *D*/2 and the temperature rise on the surface of the defective material can be rewritten as [158]:

(25) $$T\left(h,t\right)=T_{d}\left(h,t\right)+\left[T_{nd}\left(h,t\right)-T_{d}\left(h,t\right)\right]e^{-\frac{D^{2}}{16\alpha At}}$$

where *A* is the thermal diffusivity anisotropy of the composite material, which is equal to 1 for thermally isotropic materials. The exponential term in Equation (25) accounts for the physics of the diffusion of heat from the edge of the circular defect to the centre at a distance *D*/2 away. Particular attention was given by Pinto et al. [34,159] to determine the depth of embedded Teflon patches in multi-damage samples. This was accomplished by studying both magnitude (Figure 24a) and phase of thermal waves (Figure 24b). Low modulation frequencies (between 0.01 and 2 Hz) were used to observe deep lying defects and to provide information about the depth variation between multiple damage sites. 

Furthermore, in order to exploit the multifunctionality of the material in real applications, particular attention was given to the design of a connection system able to be integrated into pre-existing structures. This system needed to be characterised by high level of flexibility and a good trade-off between resolution of the analysis and power consumption. Hence, each wire of the SMA grid was connected to a circuit board in order to regulate the resolution of the thermal images acquired with the IR camera. By powering directly the circuit board with a fixed amount of power, it was possible to divert a larger amount of current through a specific portion of the sample by reducing the number of SMAs required for the analysis: by focusing on a small number of wires in a localised area the heat generated by Joule effect in that specific area increased, thus enhancing the resolution of the analysis. For this reason, smaller defects could be detected without the need to increase the total feeding current, thus keeping the total energy consumption of the system to low levels.

## 6. Conclusions

This paper presented an overview of active infrared thermography techniques recently used for the non-destructive evaluation of aerospace components. Optically stimulated thermographic methods are today the most widely used thermal imaging systems. These techniques are rapid, contactless, relatively low-cost compared to X-ray tomography and ultrasonic phased array and, when combined with advanced signal processing tools, they can successfully assess various types of material defects including flaws at the interfaces between surface coatings and their substrate, corrosion and fatigue damage in metals and debonding and delamination in composites. However, optically stimulated thermography is still not very sensitive to in-depth damage and micro-cracks with dimensions ranging from ten microns to few millimetres. Among optically stimulated thermography techniques, laser thermography is able to reveal cracks perpendicular to the sample’s surface (intralaminar), but this method is still limited to near field heating. Ultrasonic stimulated thermography provides a fast, full-filed and accurate quantification of micro-cracks due to the frictional heating caused by the interaction of ultrasonic waves with the material damage. Although this thermographic method is less affected by non-uniform heating, the ultrasonic excitation requires high-power, bulky contact transducers and may generate chaotic ultrasonic wave propagation, thus making the thermal acquisition process non-reproducible. The combination of ultrasonic stimulated thermography with the local damage resonance effect and nonlinear ultrasounds has mitigated this issue by increasing the selectivity of damage. Further quantitative studies are currently under development to enhance the capabilities of this thermal imaging method. Eddy current stimulated thermography is sensitive to a wide range of surface and sub-surface defects such as surface cracks in metals and delamination and voids in composites. Moreover, it is also able to reveal the fibre pattern in carbon fibre laminates. However, eddy current stimulated thermography is limited to near field planar defects in conductive or semi-conductive materials and is severely affected by non-uniform heating. Similarly to eddy current stimulated thermography, also microwave thermography is sensitive to superficial cracks when water is trapped in the open defect. However, this thermal method is still in its infancy for the non-destructive evaluation of aerospace structures. Indeed, the complexity of the microwave operational heating system may be a risk for the integrity of the component if high power radiation is applied. Moreover, alternative strategies to current external thermal excitation sources for composite materials, here named as material-based thermography methods, were analysed in this review. These thermographic techniques rely on the measurement of internal heating via Joule effect originated by applying electrical current either directly to main fibrous reinforcement (i.e., carbon fibres) or through the embodiment of materials such as metals inserts, carbon nanotubes and shape memory alloys. Material-based thermography methods have proved to offer a fast, low power, accurate and reliable assessment of delamination and cracks in aerospace composite components. Further work is currently under development to enhance the thermal characteristics of material enabled thermography and investigate the effects of embedded thermoresistive components on the laminate mechanical properties. 

## Figures and Tables

**Table 1:** Most common materials and types of damage used in aerospace components that are monitored with IRT.

Materials	Applications	Types of Damage Monitored with IRT
Composite laminates (e.g., CFRP and GFRP) and hybrid composites (e.g., GLARE and CARALL)	Aircraft and spacecraft primary and secondary structures	Delamination
		Impact damage and BVID
		Interlaminar disbond
		Porosity
		Fibre/Matrix Cracking
	Jet engine turbine blades	Thermal stress cracking
		Delamination between ceramic thermal barrier coating and the composite substrate
	Honeycomb panels	Skin-to-core debonding
		Facesheet delamination
		Honeycomb cell wall damage
		Absence of adhesive
		Water ingress
		Core crushing
Metals	Aircraft and spacecraft primary and secondary structures, core for honeycombs and electromagnetic wave shields	Fatigue cracks parallel and perpendicular to the surface
		Pores and voids
		Corrosion
		Material inclusions
		Welded sheets without metal diffusion (“kissing bonds”)

**Table 2:** Summary of active IRT methods used for aerospace applications.

Physical Principle	Thermal Source	Active IRT Terminology		Abbreviation
Optical Radiation	Optical flash, lamp and electrical heaters	Optically Stimulated Thermography (OST)	Pulsed Thermography [20] (or Flash Thermography)	PT
			Lock-in Thermography [21] (or Amplitude Modulated Thermography)	LIT
			Step-Heating Thermography [22] and Long Pulse Thermography [23]	SHT and LPT
			Frequency Modulated Thermography [24]	FMT
	Optical laser		Laser-spot Thermography [25] and Laser-line Thermography [26]	LST and LLT
Acoustic/Ultrasonic Wave Propagation	Acoustic/Ultrasonic horn, piezo-ceramic sensors, air-coupled transducers	Ultrasonic Stimulated Thermography [27] (or Thermosonics, Vibro-thermography and Sonic IR Thermography)		UST
		Nonlinear Ultrasonic Stimulated Thermography [28]		NUST
Electromagnetic Radiation for dielectric materials	Induced eddy currents	Eddy Current Stimulated Thermography [29]		ECST
	Microwaves	Microwave Thermography [30]		MWT
Material enabled thermo-resistive radiation for composite materials	Electrical current applied to carbon fibres	Direct Material-based Thermography (DMT)	Electrical Resistance Change Method (ERCM) coupled to thermography [3]	
	Electrical current applied to embedded steel wires	Indirect Material-based Thermography (IMT)	Metal-based Thermography [32]	MT
	Electrical current applied to embedded carbon nanotubes		Carbon Nanotubes-based Thermography [33]	CNTT
	Electrical current applied to embedded shape memory alloys wires		Shape Memory Alloys-based Thermography [34]	SMArT

**Table 3:** Summary of signal processing techniques used for OST.

Signal Processing Method	Abbreviation		Basic Principle
Differential Absolute Contrast [42,43]	DAC		It is one of the well-known processing technique that uses the difference between the temperature of a sound area and a defected area. DAC is based on the 1D solution of the Fourier heat equation.
Thermographic Signal Reconstruction [44,45]	TSR		It creates polynomial filters from log-log pre-calculations for the surface temperature response on each pixel by fitting a low-order polynomial function to the temperature evolution cooling profiles. Basic TSR significantly reduces the noise in thermal images and it can be used to generate time-derivative images without additional noise contribution. These time-derivative images can reduce the effects of non-uniform heating, the background reflection artefacts and provide good sensitivity to smaller and deeper defects. However, TSR only filters data temporally and does not make use of the spatial information.
Principal Component Thermography [46]	PCT		It uses the singular value decomposition (SVD) method to reduce an appropriately constructed matrix of observations to a set of orthogonal functions that produce a useful representation of both spatial information and temporal features for pulse thermography images. This technique provides an estimate of the average flaw depth within a flawed zone but does not provide any indication of the damage distribution. PCT is also computationally expensive.
Correlation Extraction Algorithm [47]		CEA	It is widely used in lock-in thermography and it is based on the principle that the harmonic thermal response measured by the IR camera is analysed with in-phase correlation and cross-correlation functions in order to extract information about the amplitude and phase of measured thermal signals. The feasibility of this technique depends on the length of the image sequence.
3D Normalisation Algorithm [48]		3DNA	It suppresses surface clutter conditioned by uneven heating and lateral heat diffusion for pulsed thermography. The algorithm does not require selecting a reference point for the sound area and involves the division of the IR image sequence in synthetic sequences, which are calculated by solving the corresponding three-dimensional problem of heat conduction. This method requires the determination of material thermal properties.
Multi-dimensional Ensemble Empirical Mode Decomposition [49]		MEEMD	It is used to decompose the TSR-smoothed thermal image into the high-frequency noise, low-frequency background and the informative part of the signal in order to remove the noise and non-uniform background from the thermographic data, thus improving the damage detection capabilities.
Gapped Smoothing Algorithm [50]		GSA	It is a two-dimensional reference-free quantitative detection method for sub-surface defects used for pulsed thermography. It relies on the determination of a damage index pattern that is function of the thermal contrast between real and estimated temperature profiles for all pixels. GSA method can enhance the thermal contrast and suppress the effect of non-uniform heating. It was also shown to improve the detection of damage far from the heating surface.
Partial Least Squares Thermography [51]		PLST	It is mainly used for pulsed thermography and it is based on the partial least squares regression (also known as projection to latent structures) that computes new thermal sequences that are correlated to the predicted block Y and the predictor matrix X. The matrix X corresponds to the surface temperature matrix obtained during the thermography inspection, whilst Y is defined by the observation time during which thermal images are captured. The new set of thermal images and observation time vector is composed of variables that consider only the most important signal variations. Unnecessary information present in the original thermal sequence is neglected. Contrary to PCT, PLST keeps track of the time. Hence data set can be decomposed, manipulated and recomposed, for example by omitting certain loadings with as benefits enhanced signal-to-noise ratio (SNR).
Coefficient Clustering Analysis [52]		CSA	It is a reference-based quantitative detection method based on fitting a second order polynomial model for temperature decay curves. The coefficients of the model are much less sensitive to noise and more consistent for pixels from the sound area as compared to a high order model. This technique not only provides an enhanced visual confirmation of the damage, but it also reduces the burden on the operator in post-processing data.
